# ﻿Revision of the carnivorous land snail family Streptaxidae (Stylommatophora, Achatinina) in Myanmar, with description of four new species

**DOI:** 10.3897/zookeys.1110.85399

**Published:** 2022-07-04

**Authors:** Nem Sian Man, Thanit Siriboon, Aung Lin, Chirasak Sutcharit, Somsak Panha

**Affiliations:** 1 Animal Systematics Research Unit, Department of Biology, Faculty of Science, Chulalongkorn University, Bangkok, 10330, Thailand; 2 Department of Biology, Faculty of Science, Srinakharinwirot University, Bangkok, 10110, Thailand; 3 Fauna and Flora International, No. 35, 3; 4 rd; 5 Floor, Shan Gone Condo, Myay Ni Gone Market Street, Sanchaung Township, Yangon, Myanmar

**Keywords:** Biodiversity, endemic, Fauna & Flora international, genitalia, limestone, systematics, taxonomy

## Abstract

The carnivorous terrestrial snail family Streptaxidae, recognized by having a regular to eccentric shell with complex apertural dentition, remains little-known and largely unexplored in Myanmar. This article presents historically recorded species and provides new data on this family. A total of eighteen species in five genera, namely *Carinartemis*, *Discartemon*, *Haploptychius*, *Oophana*, and *Perrottetia* from the southeastern and eastern parts of Myanmar, is examined herein. Among these, *Haploptychius* is the most diverse with eight species, while the remaining genera are comprised of fewer than five species each. *Streptaxisbirmanica* and *Streptaxisblanfordianus* are herein synonymized with *Haploptychiusblanfordi*, while *Streptaxishanleyanus* is synonymized with *Carinartemissankeyi*. Furthermore, the first genitalia and radula descriptions for three previously known species, *D.tonywhitteni*, *C.exacutus*, and *C.sankeyi*, are provided. Using comparative morphological and anatomical approaches, four new species are described: *D.paurodeviatus***sp. nov.**, *H.heliakosus***sp. nov.**, *H.tenasserimicus***sp. nov.**, and *H.karenorum***sp. nov.** This present study enhances the understanding of the land snail fauna in Myanmar, specifically the streptaxids, and highlights that limestone areas are important for biodiversity conservation.

## ﻿Introduction

Having the greatest land area in mainland Southeast Asia, Myanmar, formerly known as Burma, is recognized as having a high level of ecosystem diversity that can support a diverse variety of organisms and is part of the Indo-Burma biodiversity hotspot (Myers et al. 2000; FAO 2010; Asian Development Bank 2012). Intensive surveys of the malacofauna in Myanmar were performed in the mid-nineteenth to early twentieth centuries and were mainly conducted by American and European naturalists (see Pholyotha et al. 2020; Grego et al. 2021). However, the investigation on mollusks did not receive much attention after the independence of Myanmar in the 1940s. Fortunately, since Myanmar became more open in the 1990s, studies of several groups of Burmese land snails have gradually been published, i.e., Streptaxoidea Gray, 1860 (Páll-Gergely et al. 2020a; Sutcharit et al. 2020), Helicarionoidea Bourguignat, 1877 (Pholyotha et al. 2020; Sutcharit et al. 2020; Sutcharit and Panha 2021), Pupinidae Pfeiffer, 1853 (Páll-Gergely et al. 2015; Páll-Gergely and Grego 2019), Plectopylidae von Möllendorff, 1898 (Páll-Gergely 2018), Camaenidae Pilsbry, 1895 (Páll-Gergely et al. 2020b, 2022), and Clausiliidae Gray, 1855 (Nordsieck 2002a, b; Grego et al. 2021; Szekeres et al. 2021a, b).

The members of family Streptaxidae Gray, 1860 are known as carnivorous land snails and are characterized by an eccentric to cylindrical shell with complex apertural dentition, genitalia with hook-like structures, and living animals usually having bright yellow to red or greenish bodies (Blanford and Godwin-Austen 1908; van Benthem Jutting 1954; Berry 1963; Schileyko 2000; Verdcourt 2000; Rowson et al. 2009; Siriboon et al. 2013, 2014a, b). This group is distributed worldwide across the tropical and subtropical areas of South America, Africa, and Asia (van Bruggen 1967; Schileyko 2000; Sutcharit et al. 2010; Rowson et al. 2010). As in many predator-prey relationships, most streptaxid taxa are abundant in limestone microhabitats where their preferred food items are also abundant (Siriboon et al. 2013, 2014a, b; Inkhavilay et al. 2016). In Southeast Asia, karsts cover an area of approximately 400,000 square kilometers and are known to host high levels of animal diversity and endemism (Clements et al. 2006). Currently, ten genera and approximately 140 nominal species of streptaxid snails are recognized in the region (Blanford and Godwin-Austen 1908; van Bruggen 1972; Richardson 1988; Schileyko 2000; Siriboon et al. 2013, 2014a, b; Do and Do 2015; Inkhavilay et al. 2016; MolluscaBase 2021).

In Myanmar, the most recent reports on the superfamily Streptaxoidea (Streptaxidae and Diapheridae Panha & Naggs, 2010) were published more than one hundred years ago (see Blanford and Godwin-Austen 1908). While only a few land snail groups have been systematically revised in Myanmar (Páll-Gergely et al. 2020a; Sutcharit et al. 2020), the systematic revision of the streptaxid snails in several other countries of mainland Southeast Asia (i.e., Laos, Vietnam, and Thailand) has advanced during the past decade (Siriboon et al. 2013, 2014a, b; Inkhavilay et al. 2016; Do 2017; Bui et al. 2019; Siriboon et al. 2020; Sutcharit et al. 2020).

During land snail expeditions in Myanmar in 2015 and 2016, streptaxid species were discovered in several localities in southeastern (Mon State, Kayin State, and Tanintharyi Region) and eastern (Shan State and Mandalay Region) parts of Myanmar under the cooperation of the Forest Department of Myanmar (FDM), Fauna & Flora International (FFI), and Chulalongkorn University. Herein, we discuss the taxonomy of the carnivorous terrestrial snail family Streptaxidae collected in Myanmar. The molecular phylogeny by Siriboon et al. (2020) suggests that some genera are non-monophyletic, especially *Oophana* Ancey, 1884 and *Haploptychius* von Möllendorff, 1906. However, in this study the traditional concept based on morphological characters is applied, without acknowledging their respective type species. Therefore, this research aims to 1) revise the systematics of the streptaxid species with eccentric shell shape based on the shell, radula morphology and genital anatomy (if available), especially the penial hooks; and 2) record all species of streptaxid species with an eccentric shaped shell that have been reported from Myanmar based on the literature and historical museum collections.

## ﻿Materials and methods

**Figure 1.**
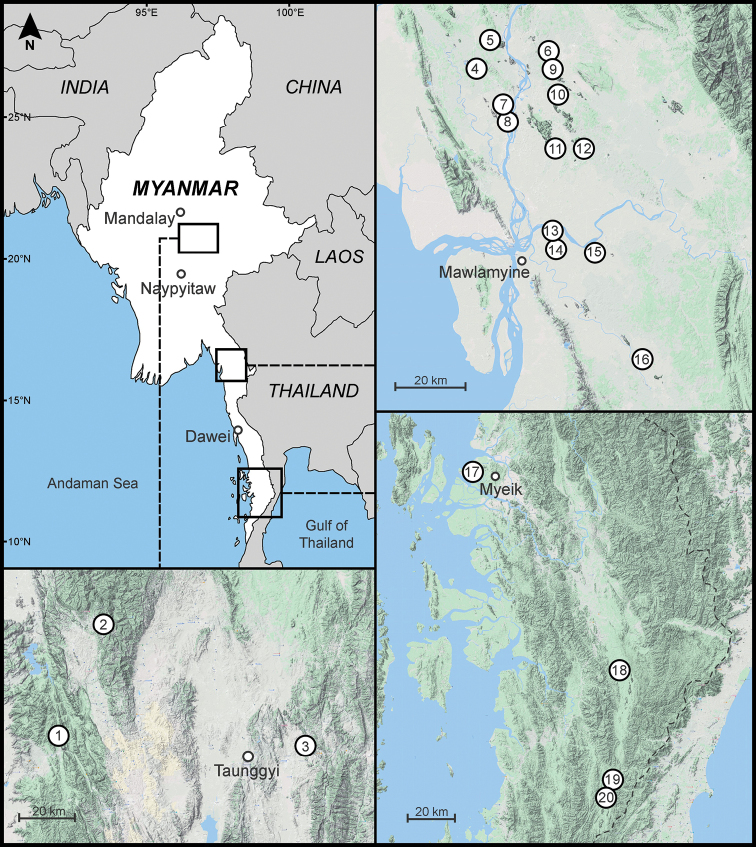
Approximate sampling locations used in this study. The location name and coordinates are listed in Table 1. Historical type localities of a broad scale, such as Tavoy, Burma, Moulmein, Mergui, and Tenasserim, are not indicated on this map.

Streptaxid specimens were collected during 2015 and 2016 by the Animal Systematics Research Unit (ASRU) members, Department of Biology, Faculty of Science, Chulalongkorn University, Bangkok, Thailand. These field surveys were conducted under a MOU between the Forest Department, Ministry of Natural Resources and Environmental Conservation and Forestry, Myanmar and Fauna & Flora International (**FFI**) with Letter No. 0092. The field surveys focused on non-limestone and limestone areas in Shan State, Mon State, Kayin State, Mandalay Region, and Tanintharyi Region. The coordinates were recorded using GPS. Approximate collection localities are presented in Table 1 and Figure 1.

**Table 1.** Shell measurements of streptaxid species from Myanmar recognized in this study. Numbers listed before collection locality correspond to those in the map in Fig. 1.

Species, localities and CUMZ no.	Number of specimens	Ranges, mean ± S.D. in mm.			Number of whorls
		Shell height	Shell width	H/W ratio	
*Discartemontonywhitteni*					
20. Phra (Buddha) Cave, Tanintharyi: (13001)	7	3.4–5.1 4.5±0.64	9.5–12.7 11±1.05	0.27–0.5 0.4±0.08	6–6½
*Discartemonpaurodeviatus* sp. nov.					
17. Phataw Phatet Island, Tanintharyi: (13002, 13003, 13004)	17	6.8–8.1 7.5±3.48	10.8–12 11.3±0.35	13.8–16.8 1.5±0.07	6–6½
*Oophanamouhoti*					
19. Phra (Buddha) Cave, Tanintharyi: (13005)	1	11.2	10	0.6	6½
*Perrottetiatheobaldi*					
3. Aik Kham Cave, Shan: (13006)	4	3.6–4.2 3.8±0.31	5.6–5.8 5.7±0.10	1.4–1.6 1.5±0.10	5–5½
*Haploptychiussolidulus*					
16. Pathen mountain, Hpa-an, Kayin: (13007)	15	10.8–12.4 11.6±0.61	11.3–12.6 11.8±0.46	0.9–1.1 1.02±0.03	6–6½
*Haploptychiusthebawi*					
1. Pyinyaung Village, Meiktila, Mandalay Region (Apache Cement Co. Ltd): (13008)	4	7.5–9.0 7.8±0.69	7.5–8.7 8.1±0.51	0.8–1.1 1.03±0.13	6–6½
2. Lin Way Monastery, Ywangan, Shan: (13009)	7	6.7–7.3 7.1±0.24	6.5–7.9 7.2±0.42	0.9–1.2 1.02±0.08	5½–6
3. Aik Kham Cave, Taunggyi, Shan: (13010)	3	4.6–5.3 5.0±0.36	5.0–5.7 5.4±0.37	1.1–1.2 1.08±0.03	6–6½
*Haploptychiustenasserimicus* sp. nov.					
18. Lampane Cave, Tanintharyi: (13011, 13012)	5	4.1–5.0 4.5±0.36	8.1–9.7 8.7±0.59	1.7–2.0 1.9±0.08	6
*Haploptychiusheliakosus* sp. nov.					
9. Bardai Mountain, Hpa-an, Kayin: (13013, 13014, 13015)	39	9.7–11.6 10.6±0.60	7.3–10.3 9.1±1.05	0.6–0.9 0.8±0.10	7–7½
6. Kyonknow Cave, Hpa-an, Kayin: (13016)	7	8.4–9.9 9.2±0.61	8.1–9.9 8.9±0.57	0.8–1.2 0.9±0.10	6½–7
*Haploptychiuskarenorum* sp. nov.					
10. Waiponla Mountain, Hpa-an, Kayin: (13017, 13018)	6	11.4–12.4 11.8±0.40	8.2–10.0 8.9±0.70	0.6–0.8 0.7±0.08	6–7½
5. Taung Lay Cave, Hpa-an, Kayin: (13019)	2	8.8–9.7 9.3±0.4	10.5–12 11.3±0.8	1.14–1.36 1.22±1.10	6½–7
*Carinartemisexacutus*					
11. Sadhdan Cave, Hpa-an, Kayin: (13020)	3	6.2–7.3 6.7±0.45	13.0–14.0 13.03±0.4	1.8–2.0 1.9±0.15	6–6½
4. Bayin Nyi Cave, Hpa-an, Kayin: (13021, 13022)	23	6.3–6.8 6.6±0.22	6.8–12.6 12.2±0.4	1.7–2.0 1.8±0.09	6–6½
12. Lun Nga Mountain, Hpa-an, Kayin: (13023, 13024)	64	5.5–6.3 5.9±0.33	13.2–13.9 13.5±0.28	2.1–2.5 2.2 ±0.16	5½–6
7. Taung Wine Cave, Hpa-an, Kayin: (13025, 13026)	9	7.4–7.5 7.5±0.05	12.0–13.1 12.5±0.56	1.6–1.7 1.7±0.89	6–6½
8. Kaw Ka Taung, Hpa-an, Kayin: (13027)	1	7.6	12	1.6	6½
*Carinartemissankeyi*					
14. Saddan Cave, Mawlamyine, Mon: (13028)	7	6.9–8.3 7.6±0.51	9.0–10.5 9.7±0.55	1.2–1.4 1.3±0.11	6–7
13. Kayon Cave, Mawlamyine, Mon: (13029, 13030)	18	7.0–8.2 7.5±0.49	9.1–10.2 9.7±0.49	1.2–1.4 1.2±0.07	6½–7
15. Dhammatat Cave, Mawlamyine, Mon: (13031)	12	5.5–7.4 6.5±0.57	10.4–11.6 11.1±0.44	1.5–2.0 1.7±0.15	6–7

For anatomical studies, living specimens were photographed, euthanized following the protocol by the American Veterinary Medical Association (2020), and then preserved in 70% ethanol (v/v). Taxonomic identifications were made based on Kobelt (1905–1906), Blanford and Godwin-Austen (1908), Schileyko 2000; Siriboon et al. (2013, 2014a, b), Inkhavilay et al. (2016), and Sutcharit et al. (2020). Shell height, shell width and whorl count were measured, and the height/width ratio was calculated following Siriboon et al. (2013). The shells were photographed using a Nikon camera with a Nikon 105 Macro lens. One to five adult specimens in ethanol were dissected, and the genitalia were examined under a stereomicroscope. A representative specimen was illustrated with the aid of a camera lucida. The buccal masses were removed, and the radulae were soaked in 10% (w/v) NaOH, and then cleaned in distilled water. Radula, penial hooks, and vaginal hooks were examined and photographed using scanning electron microscopy (**SEM**; JEOL, JSM-5410 LV).

All the nominal species names described as new to science in this work are attributed to the first and last authors (Man & Panha). Thus, a complete citation of the authors is Man & Panha in Man et al.

### ﻿Anatomical abbreviations

**ag**, albumen gland; **at**, atrium; **fo**, free oviduct; **gd**, gametolytic duct; **gs**, gametolytic sac; **hd**, hermaphroditic duct; **ov**, ovary; **p**, penis; **pr**, penial retractor muscle; **ps**, penial sheath; **psr**, penial sheath retractor muscle; **sv**, seminal vesicle; **ta**, talon; **v**, vagina; **vd**, vas deferens (Siriboon et al. 2013, 2014a, b; Sutcharit et al. 2010).

### ﻿Institutional abbreviations

Materials examined in this study were deposited in the following institutions:

**CUMZ**Chulalongkorn University Museum of Zoology, Bangkok;

**MCZ**Museum of Comparative Zoology, Harvard University, Massachusetts;

**MNHN**Muséum National ďHistoire Naturelle, Paris;

**NHM**Natural History Museum, London;

**NHMUK** when citing specimens deposited in the NHM;

**SMF**Forschungsinstitut und Naturmuseum Senckenberg, Frankfurt am Main.

## ﻿Systematics

### ﻿Family Streptaxidae Gray, 1860

#### ﻿Genus Discartemon

Taxon classificationAnimaliaStylommatophoraStreptaxidae

Pfeiffer, 1856

DE6A3DA2-77E1-501F-9C11-F63C18280F20

Discartemon Pfeiffer, 1856: 173. Ancey 1884: 399. Tryon 1885: 58. Gude 1903: 226. van Benthem Jutting: 1954: 71–94. Zilch 1960: 560. Richardson 1988: 182–185. Schileyko 2000: 784. Siriboon et al. 2014a: 48, 49. Inkhavilay et al. 2016: 25. Sutcharit et al. 2020: 151.
Odontartemon (Discartemon) – Kobelt 1905: 91, 96.

##### Type species.

*Streptaxisdiscus* Pfeiffer, 1853, by subsequent designation by Ancey (1884: 399).

##### Diagnosis.

Shell flattened to globose-heliciform. Last whorl rounded to angular, less distorted, and often with peripheral keel; whorls regularly to rapidly expanded. Apertural dentition varied, ranging from only one parietal lamella to additional dentition: upper palatal, palatal, basal, columellar and supracolumellar lamellae. Genitalia with short to long penis, sometimes with penial appendix and penial sheath covering entire penis length. Penial hooks present and vaginal hook absent.

##### Remarks.

This genus was recently revised in both shell and genital diagnostic characters, and the intrageneric relationship was discussed (Siriboon et al. 2014a, 2020). At present, the genus *Discartemon* comprises thirty recognized species, including a new species described herein (Sutcharit et al. 2020).

#### ﻿ Discartemon tonywhitteni

Taxon classificationAnimaliaStylommatophoraStreptaxidae

Sutcharit & Panha, 2020

AB2F805A-63B0-5F7C-B8D4-2D85333E6E7A

**Figure 2.**
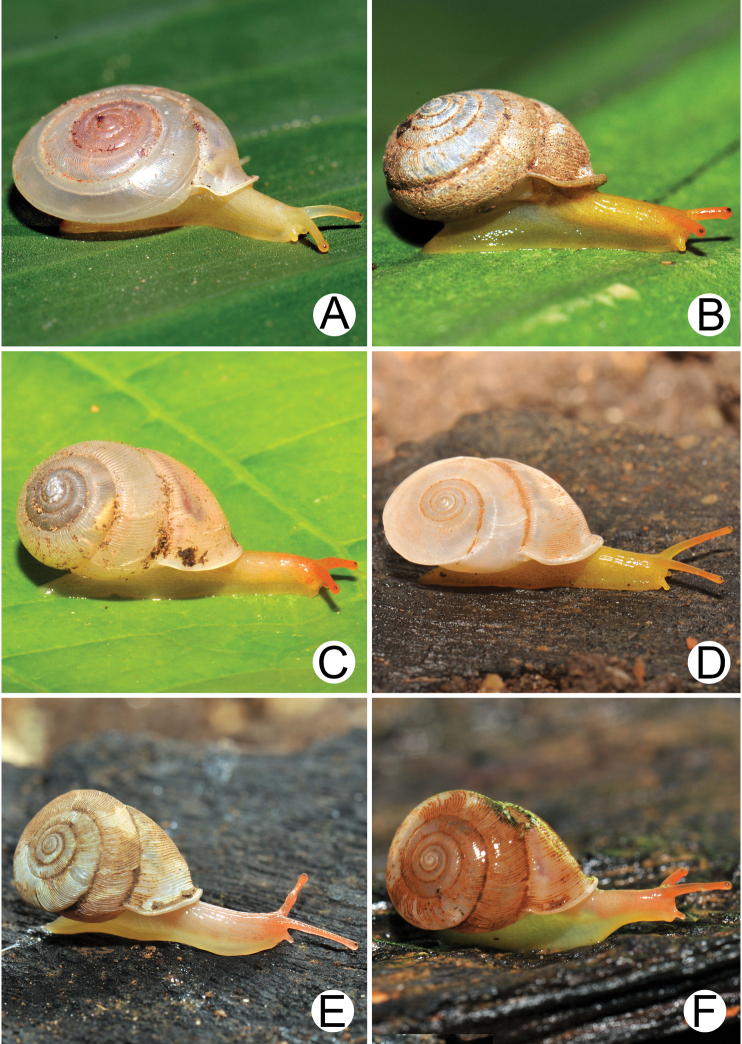
Living snails **A***Discartemontonywhitteni* from near Phra (Buddha) Cave, Tanintharyi Region (shell width ~ 10 mm) **B***Discartemonpaurodeviatus* sp. nov. from the type locality (shell width ~ 11 mm) **C***Haploptychiusheliakosus* sp. nov. from the type locality (shell height ~ 9 mm) **D***Carinartemisexacutus* from Lun Nga Mountain, Kayin State (shell height ~ 14 mm) **E, F***Carinartemissankeyi***(E)** from Kayon Cave, Mon State (shell height ~ 10 mm) and **(F)** from Saddan Cave, Mawlamyine, Mon State (shell height ~10 mm).

**Figure 3.**
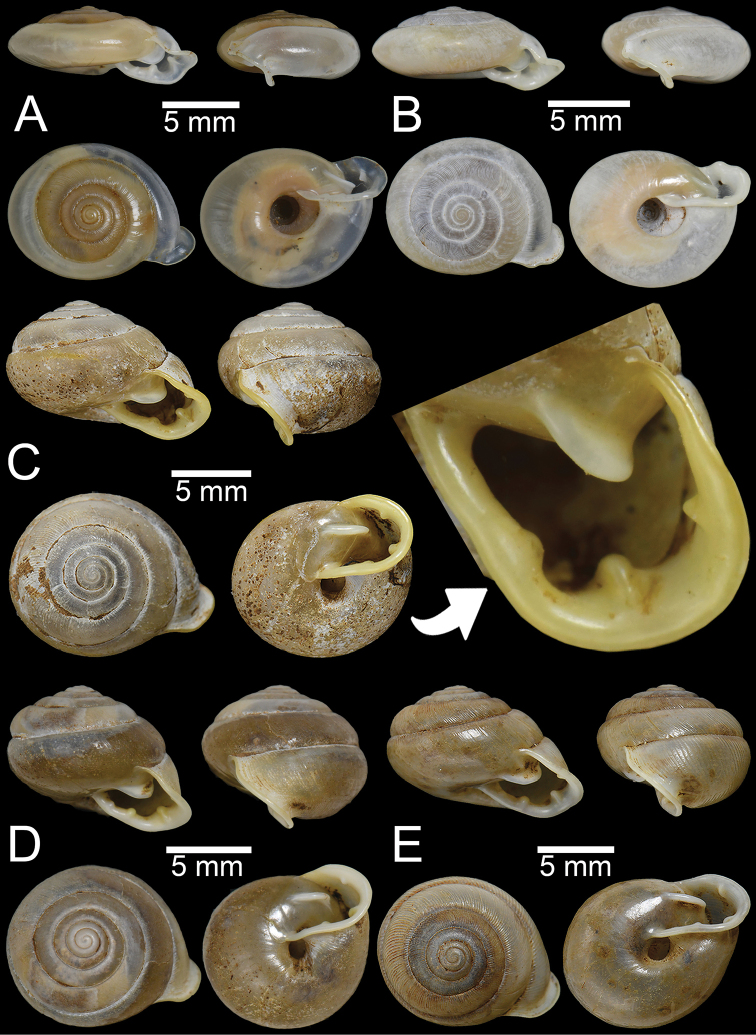
**A, B***Discartemontonywhitteni*, specimen CUMZ 6264 from Phra (Buddha) Cave, Tanintharyi Region **C–E***Discartemonpaurodeviatus* sp. nov. **C** holotype CUMZ 13002 with apertural dentition and **D, E** paratypes CUMZ 13003 from the type locality.

**Figure 4.**
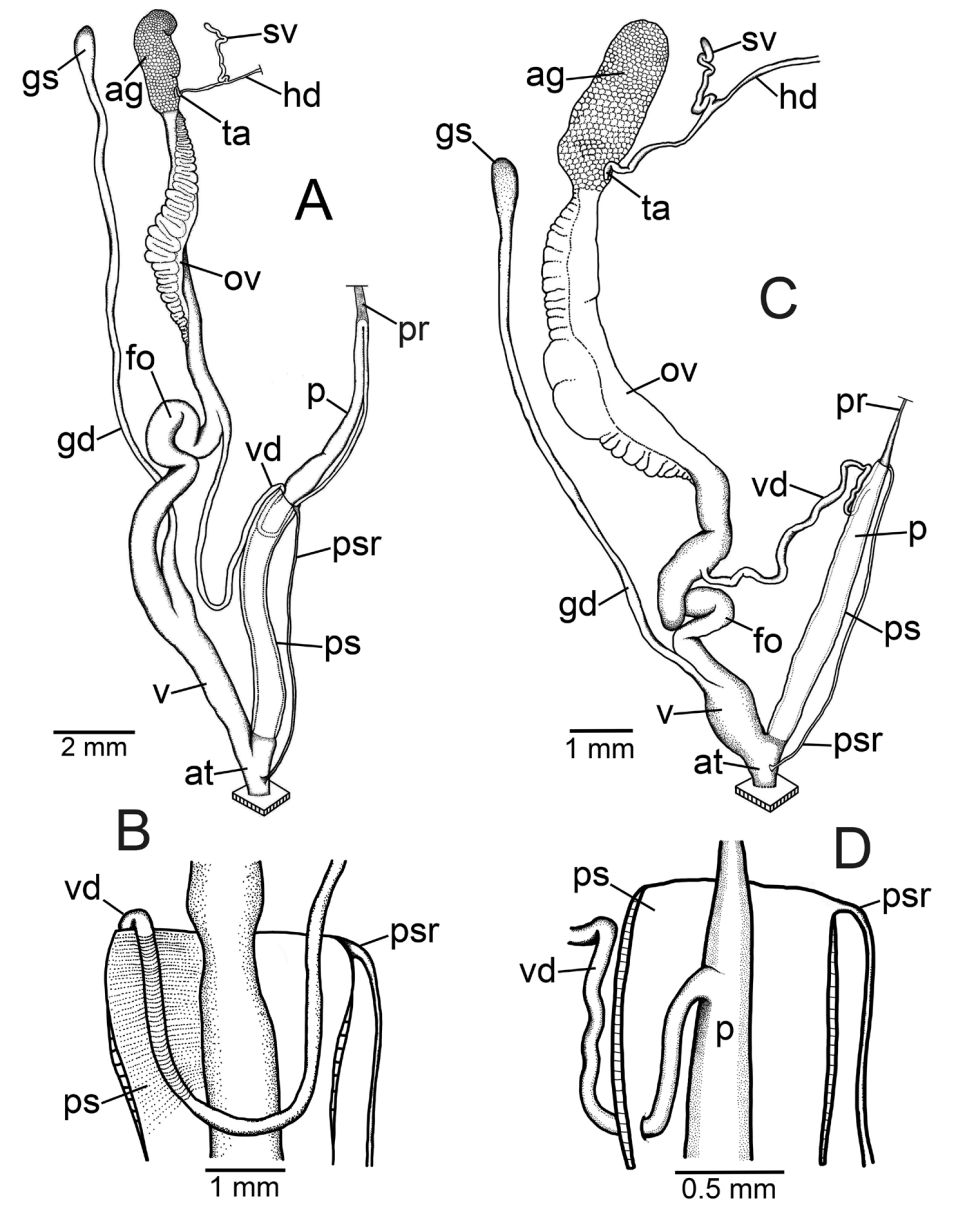
Genital anatomy of **A, B***Discartemontonywhitteni*, specimen CUMZ 13001 **A** reproductive system and **B** insertion of vas deferens into penial sheath **C, D***Discartemonpaurodeviatus* sp. nov., paratype CUMZ 13003 **C** reproductive system and **D** insertion of vas deferens into penial sheath.

**Figure 5.**
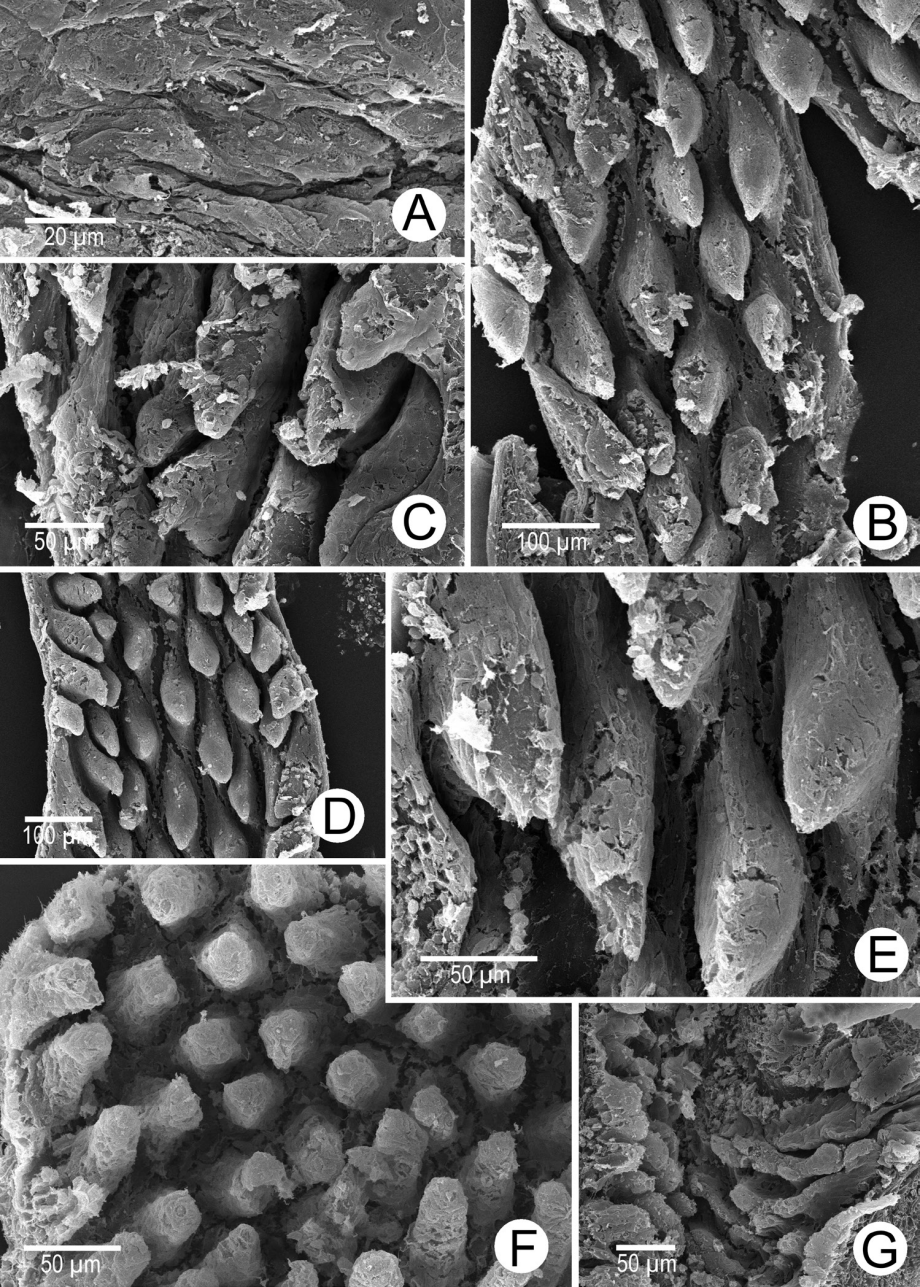
Internal sculpture of genitalia of *Discartemontonywhitteni*, specimen CUMZ 13001 **A** atrium surface **B** arrangement of penial hooks on proximal part of penis **C** lateral view of proximal penial hooks **D** arrangement of penial hooks on middle part of penis **E** high magnification view of pointed penial hooks on middle part of penis **F** top view of distal penial hooks embedded in penial papillae **G** arrangement of transverse vaginal folds.

Figs 1 , 2A , 3A, B , 4A, B , 5 , 24A

Discartemon tonywhitteni Sutcharit & Panha in Sutcharit et al. 2020: 154, fig. 3a–c. Type locality: Phra (Buddha) Cave, Lenya National Park, Tanintharyi Township, Tanintharyi Region, Myanmar.

##### Material examined.

***Holotype***CUMZ 5108; ***paratypes***CUMZ 5107 (14 shells), CUMZ 5101 (30 shells), CUMZ 5102 (25 shells), CUMZ 5104 (18 shells), CUMZ 5105 (30 shells), CUMZ 5106 (20 juvenile shells). Limestone outcrop ~ 3.4 km south of the Phra (Buddha) Cave, Lenya National Park, Tanintharyi Township, Tanintharyi Region, Myanmar (11°11'56.2"N, 99°10'25.7"E): CUMZ 13001 (7 specimens in ethanol; Fig. 3A, B).

##### Diagnosis.

This species was clearly described in Sutcharit et al. (2020). Shell is depressed-heliciform, white and translucent, spire low conical to convex with wide and depressed suture. Shell surface glossy, with fine transverse ridges that diminish below periphery; varices present. Embryonic shell large, ~ 2½ whorls with smooth surface; following whorls in regularly expanding coil. Last whorl usually angular; widely open umbilicus. Apertural dentition with one strong parietal, one palatal, one basal, one columellar and one supracolumellar lamella (Fig. 3A, B).

***Genital organs*.** Atrium (at) short. Penis (p) long and slender. Penial sheath (ps) thin and extending ~1/2 to 3/4 of penis length; penial sheath retractor muscle (psr) very thin, originating at atrium and inserting distally on penial sheath (Fig. 4A). Vas deferens (vd) very short, ~ 1/6 of penial sheath length and passes through penial sheath before entering penis distally (Fig. 4B). Penial retractor muscle (pr) thin and very long, inserting at penis and vas deferens junction.

Internal wall of atrium generally smooth (Fig. 5A). Proximal and middle penial wall with dense and translucent penial hooks (~ 5 hooks/200 μm^2^) and located on conical papillae (Fig. 5B–D). Penial hooks small (<0.04 mm in length), expanded at base, tips pointed (Fig. 5C, E). and curved towards genital orifice. Most penial hooks with blunt tips (Fig. 5E). Distal penial wall with dense and translucent conical penial papillae with embedded penial hooks, ~ 12 penial papillae/200 μm^2^ (Fig. 5F).

Vagina (v) short, ~ 1/3 of penis length. Gametolytic duct (gd) a long tube extending as far as albumin gland; gametolytic sac (gs) ovate. Free oviduct (fo) long, ~ 2 times of vagina length. Oviduct (ov) slender and folded; prostate gland inconspicuous and bound to oviduct. Talon (ta) small, short and club shaped. Hermaphroditic duct (hd) bearing long seminal vesicle (sv) ca. twice as long as the length from talon to branching point of seminal vesicle (Fig. 4A).

Vaginal wall with transverse vaginal folds; vaginal hook absent (Fig. 5G).

***Radula*.** Each row consists of ~ 35‒49 teeth with formula (24‒17) ‒1‒ (17‒24). Central tooth very small with pointed cusp. Lateral and marginal teeth undifferentiated, unicuspid and lanceolate. Latero-marginal teeth gradually reducing in size, with outermost teeth much smaller and shorter than inner teeth (Fig. 24A).

##### Distribution.

*Discartemontonywhitteni* is the southernmost distributed species and, currently, is known as the only form from the type locality (Fig. 1), which is an isolated limestone outcrop in Tanintharyi Region, southern Myanmar (Sutcharit et al. 2020).

##### Remarks.

Sutcharit et al. (2020) described *D.tonywhitteni* based only on shells. Fortunately, seven alcohol-preserved specimens collected near the type locality were sent by the FFI staff after the species was published. These additional specimens allow us to describe the genitalia and radular morphology of this species herein.

#### ﻿ Discartemon paurodeviatus

Taxon classificationAnimaliaStylommatophoraStreptaxidae

Man & Panha sp. nov.

C970CA87-3375-5ED1-8FDA-CF993AD6D08A

https://zoobank.org/7F0B6632-7902-483B-A580-A3900EB06D6E

**Figure 6.**
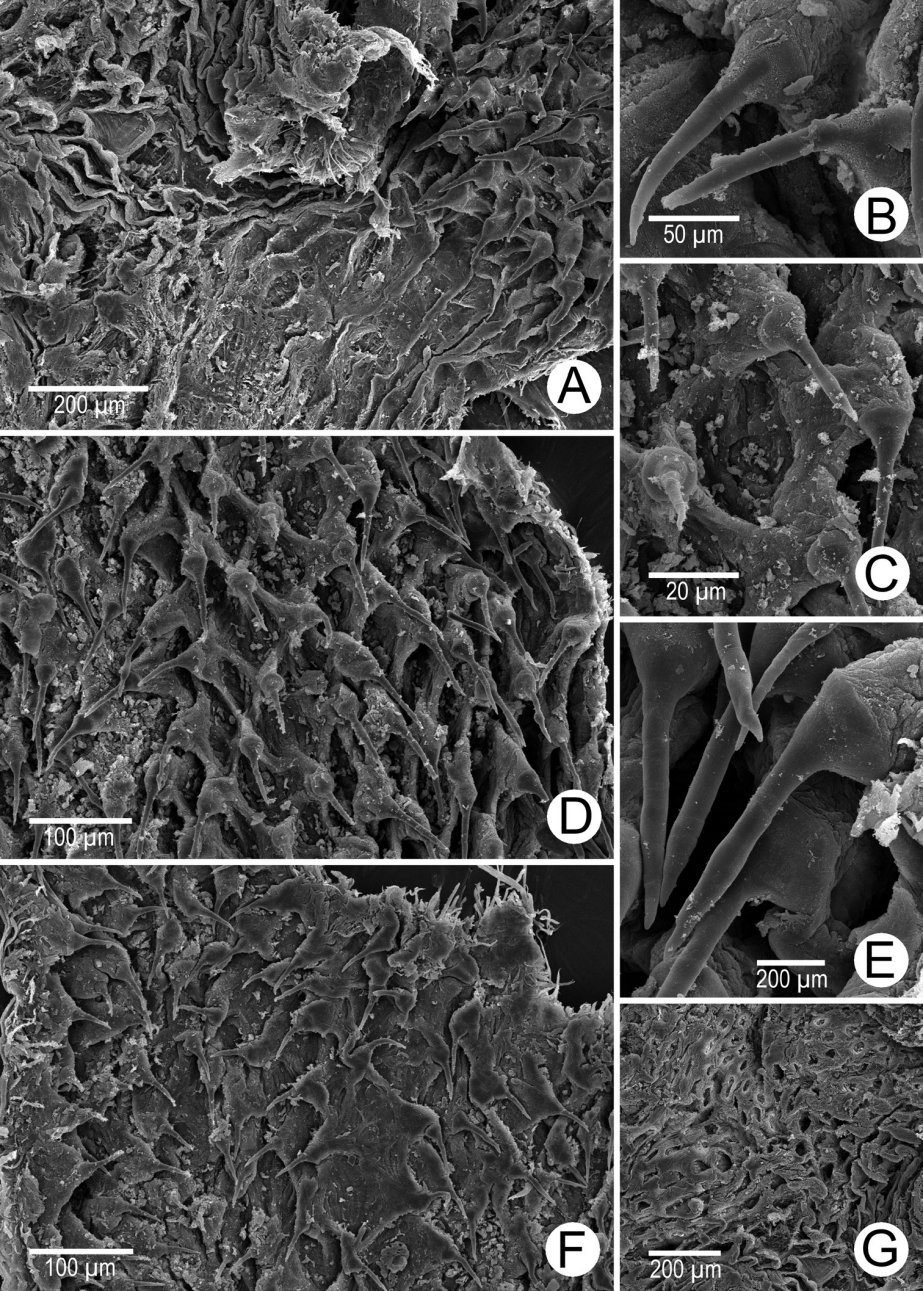
Internal sculpture of genitalia of *Discartemonpaurodeviatus* sp. nov., paratype 13003 **A** junction of atrium, vagina, and proximal penis **B, C** lateral view of penial hooks on proximal part of penis **D** reticulated arrangement of penial hooks on middle part of penis **E** lateral view of penial hooks **F** arrangement of penial hooks on distal part of penis **G** arrangement of thickened reticulated vaginal folds.

Figs 1 , 2B , 3C–E , 4C, D , 6 , 24B

##### Type material.

***Holotype***CUMZ 13002 (Fig. 3C). Measurements: shell height 7.6 mm, shell width 11.0 mm, and 6 whorls. ***Paratypes***CUMZ 13003 (14 shells; Fig. 3D, E), CUMZ 13004 (3 specimens in ethanol), NHMUK (2 shells), all from the type locality.

##### Type locality.

Small hill on Pahtaw Pahtet Island (~ 500 m west of Myeik Town), Myeik Township, Tanintharyi Region, Myanmar (12°26'3.5"N, 98°35'14.1"E).

##### Diagnosis.

*Discartemonpaurodeviatus* sp. nov. can be distinguished from *D.collingei* (Sykes, 1902) from Malaysia by having a larger shell, rounded last whorl, semi-ovate and slightly reflected aperture, and sometimes supracollumellar lamellae are present. Compared with *D.vandermeermohri* van Benthem Jutting, 1959 and *D.mekalostraka* Siriboon & Panha, 2014 from Thailand, this new species has a slightly extended last whorl from the penultimate whorl, last whorl slightly axially deflected, widely open umbilicus, and semi-ovate aperture. The genital organs of *D.paurodeviatus* sp. nov. differ from *D.mekalostraka* by having corrugated atrium without pore, short penis, dense brownish and long slender penial hooks located on penial papillae, and short vagina with thickened and reticulated vaginal folds.

##### Description.

Shell globose-heliciform, white and translucent; whorls 6–6½; spire conical with distinct suture. Shell surface glossy with fine transverse ridges that diminish below periphery; varices present. Embryonic shell ~ 2½ whorls with smooth surface; following whorls regularly coiled. Last whorl rounded, little axially deflected and extruded from the penultimate whorl. Aperture semi-ovate; peristome yellowish to whitish, discontinuous, thickened, expanded, and slightly reflected. Apertural dentition with one strong parietal, one small palatal, one large basal, and one small columellar lamella; sometimes with small supracolumellar lamellae. Umbilicus open and deep (Fig. 3C–E).

***Genital organs*.** Atrium (at) short. Penis (p) long and slender. Penial sheath (ps) very thin, extending entire penis length; penial sheath retractor muscle (psr) very thin, originating at atrium and inserting distally on penial sheath (Fig. 4C). Vas deferens (vd) passes through penial sheath without insertion before entering penis distally (Fig. 4D). Penial retractor muscle (pr) thin and long, inserting at penis and vas deferens junction.

Internal wall of atrium corrugated with sparse atrial pores (Fig. 6A). Penial wall with scattered, very long, slender, and pale brown penial hooks (~ 10 hooks/200 μm^2^), hooks located on conical papillae that are separated by distinct reticulated folds (Fig. 6C, D). Penial hooks of small size (< 0.08 mm in length), slightly expanded and conical at base, tip sharp and directed towards genital orifice (Fig. 6B, E, F).

Vagina (v) very short and stout, ~ 1/7 of penis length. Gametolytic duct (gd) a long and slender tube extending as far as albumin gland; gametolytic sac (gs) ovate. Proximal free oviduct (fo) convoluted and distally long and thick, ~ 3 times of vagina length. Oviduct (ov) enlarged and folded; prostate gland inconspicuous and bound to oviduct. Talon (ta) small, short and club shaped. Hermaphroditic duct (hd) bearing short and thickened seminal vesicle (sv) and ca. same length as from talon to branching point of seminal vesicle (Fig. 4C).

Vaginal wall generally with thickened reticulated vaginal folds, smooth surface, and vaginal pores present (Fig. 6A, G).

***Radula*.** Each row consists of ~ 21‒33 teeth with formula (16‒10)‒1‒(10‒16). Central tooth small with pointed cusp. Lateral and marginal teeth largest and undifferentiated, unicuspidal, and lanceolate. Latero-marginal teeth rapidly reducing in size, with outermost teeth much smaller and shorter than inner teeth (Fig. 24B).

##### Etymology.

The specific name *paurodeviatus* is derived from the Greek word *pauros* meaning little or few and the Latin word *devius* meaning out of the way. It refers to the last whorl of the new species as being slightly axially deflected and extruded from the penultimate whorl.

##### Distribution.

The species is only known from the type locality in southern Myanmar (Fig. 1).

##### Remarks.

Variation occurs in the possession of supracolumellar lamellae in some specimens. Currently, two species of *Discartemon* (*D.paurodeviatus* sp. nov. and *D.tonywhitteni*) have been recognized from Myanmar (Sutcharit et al. 2020). These two species are noticeably different in shell morphology and internal structure of genitalia and found in distant localities from each other.

#### ﻿Genus Oophana

Taxon classificationAnimaliaStylommatophoraStreptaxidae

Ancey, 1884

AE89D003-D2F9-56F8-B23C-DB8628D45A4D

Oophana Ancey, 1884: 508. Tryon 1885: 58. Richardson 1988: 233. Schileyko 2000: 796. Clements 2006: 125.
Odontartemon (Oophana) – Kobelt 1906: 91, 101. Thiele 1931: 730. van Benthem Jutting 1954: 95. Zilch 1960: 562.

##### Type species.

*Enneabulbulus* Morelet, 1862 by subsequent designation by Kobelt (1906: 101).

##### Diagnosis.

Shell globosely ovate to sub-heliciform, translucent to opaque. Penultimate whorl slightly extended beyond last whorl. Last whorl rounded or flattened along the periphery, and axially deflected from penultimate whorl. Aperture oblique-ovate to squarish; apertural dentition with parietal (one or two), palatal, basal, and columellar lamellae. Genitalia with penial sheath covering ~ 1/2 of penis length, and penial hook present.

##### Remarks.

The genus *Oophana* shares the ovate shell shape with the *Haploptychius*. However, *Oophana* can be distinguished by generally having a greater number of apertural dentitions (parietal, palatal, basal and columellar lamellae), while *Haploptychius* has only one parietal lamella (Schileyko 2000; Inkhavilay et al. 2016).

Generally, despite *Oophana* and *Indoartemon* Forcart, 1946 possessing an ovate shell with a blunt spire, the former can be distinguished by its parietal, palatal, basal, and columellar lamellae, while the latter possesses only one parietal and one palatal lamella (Schileyko 2000; Siriboon et al. 2014a). *Oophana* can be distinguished from *Discartemon* and *Perrottetia* Kobelt, 1905 by its globosely ovate shell, axially deflected last whorl, and penultimate whorl more or less extended from the last whorl. In comparison, *Discartemon* has a flattened to a globose-heliciform shell, regularly to rapidly growing last whorl that is less deflected, and penultimate whorl does not extend from the last whorl (Siriboon et al. 2014a). *Perrottetia* possesses a sub-heliciform and depressed shell, with longitudinal furrows behind the apertural lip, and a less deflected last whorl (Siriboon et al. 2013; Inkhavilay et al. 2016). Moreover, *Oophana* can easily be separated from the *Carinartemis* Siriboon & Panha, 2014 by their rounded penultimate whorl with more apertural dentition, whereas the latter genus has a sharply keeled penultimate whorl, and without or with only one parietal lamella (Siriboon et al. 2014b).

Generally, *Oophana* shows high variability in shell form, and apertural dentition with upper palatal and supracolumellar lamellae occurring in some species (Siriboon et al. 2020). However, examination of the genitalia in most species including the type species is still limited, making comparisons with other congeners difficult.

The phylogenetic relationships of the *Oophana* s.l. from Thailand were recently shown to be polyphyletic and comprised of three groups. These polyphyletic groups could possibly be recognized as distinct genera supported by their unique shell and genital characters (Siriboon et al. 2020). Furthermore, the oblique-heliciform shell and apertural dentition with four lamellae are shared among these three polyphyletic groups. Nevertheless, without the information on the type species, these characters are insufficient to restrict the true *Oophana* s.s., and so, in this revision we place the streptaxids that have an oblique-ovate shell and apertural dentition with four lamellae within the genus *Oophana* s.l.

#### ﻿ Oophana elisa

Taxon classificationAnimaliaStylommatophoraStreptaxidae

(Gould, 1856)

553A1999-7F0F-5F20-BDD7-B2BAB4A2B46B

Streptaxis elisa Gould, 1856: 13. Type locality: an island in the Mergui Archipelago. Pfeiffer 1868: 448. Blanford and Godwin-Austin 1908: 14.
Odontartemon elisa – Kobelt 1906: 126.
Perottetia elisa – Kobelt 1910: 151.
Oophana elisa – van Benthem Jutting 1954: 96. Richardson 1988: 234, 235. Clements 2006: 126.

##### Remarks.

The shell is oblique-heliciform with convex spire and a distinct suture. Whorls 7 and shell surface with transverse ridges that diminish below the periphery. Shell periphery is keeled, and last whorl axially deflected. Umbilicus open and deep. Aperture is subquadrangular, peristome reflected. Apertural dentition with one parietal, one basal, two weak palatal, and one columellar lamella.

This species is known only from the type locality, in the southern part of Myanmar. No fresh materials were collected in this survey. Herein, the description of this species is based only on the original description. Gould (1856) received the specimen from Rev. J. Benjamin and described it without providing any figures. In the description, Gould compared *O.elisa* with two species: *Seychellaxissouleyetianus* (Petit, 1841) and *Haploptychiuspyriformis* (Pfeiffer in Philippi, 1845). In differentiating the three, Gould noted that *O.elisa* has a larger and a more depressed shell than *S.souleyetianus*, and that *O.elisa* has a more elongated shell that is two-fold larger in size and posterior denticle (presumably parietal lamella) smaller than *H.pyriformis*. Based on this comparison, we assume that the shell of *O.elisa* differs from all other known congeners from Myanmar and Peninsular Malaysia by its largely deflected last whorl, discoid spire and keeled penultimate whorl. Besides probably having the same distribution range, *O.elisa* is similar to *D.paurodeviatus* sp. nov. in having a quite straight and parallel aperture lip, and four apertural dentitions. However, *O.elisa* seems to have an oblique-ovate shell with more deflected last whorl, keeled penultimate whorl, and discoid spire. Meanwhile, *D.paurodeviatus* sp. nov. possesses a globose shell with less deflected last whorl, and penultimate whorls being rounded. To date, no specimens or illustrations have been found after the first record.

#### ﻿ Oophana mouhoti

Taxon classificationAnimaliaStylommatophoraStreptaxidae

(Pfeiffer, 1863)

785DF7C4-D124-55A3-9277-FBDA37ECA0AF

Figs 1 , 7A, B

Streptaxis mouhoti Pfeiffer, 1863 [1862]: 273. Type locality: Siam [Thailand]. Martens 1867: 84, pl. 22, fig. 22. Pfeiffer 1868: 446. Nevill 1878: 4. Tryon 1885: 80, pl. 15, fig. 46. Bourguignat 1889: 133.
Streptaxis johswichi Martens, 1864: 528. Type locality: Siam, bei Petchaburi [Petchaburi Province, Thailand].
Streptaxis mouhoti var. johswichi – Martens 1864: 528. Pfeiffer 1868: 446.
Gonaxis mouhoti – Bourguignat 1889: 133.
Odontartemon (Oophana) mouhoti – Kobelt 1906: 104, 105, pl. 55, fig. 23.
Oophana mouhoti – Richardson 1988: 235, 236. Siriboon et al. 2020: 5, 14, 15.

##### Material examined.

**Figure 7.**
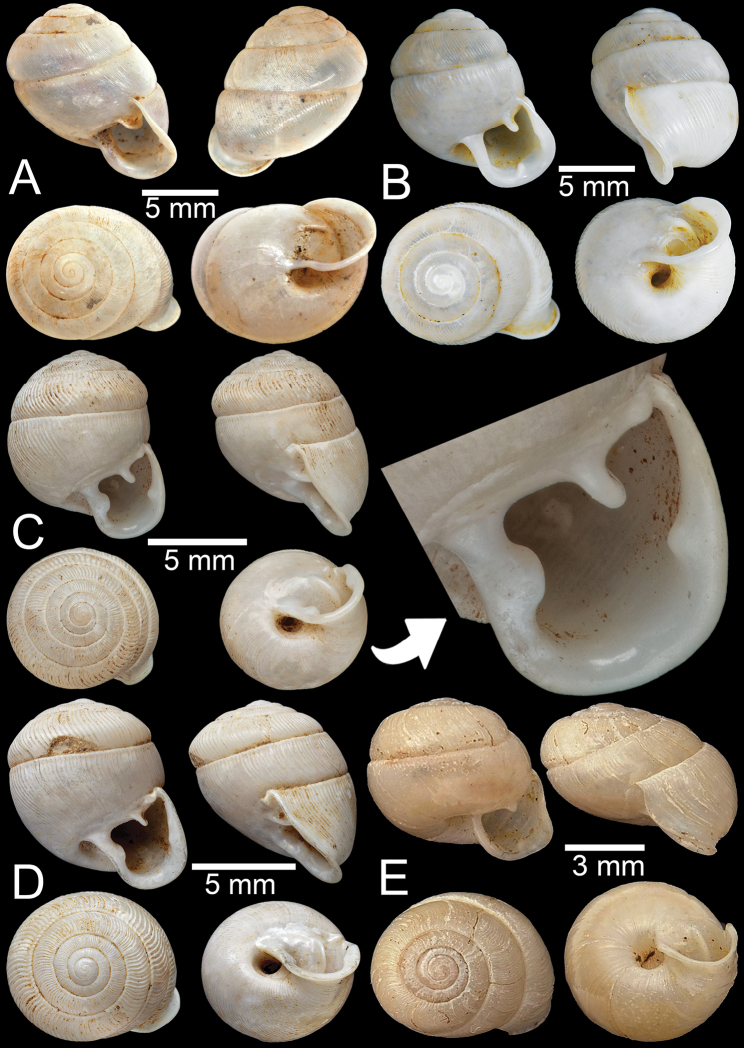
**A, B***Oophanamouhoti***A** possible syntype NHMUK ex. Cuming collection from Camboja and **B** specimen CUMZ 13005 from Phra (Buddha) Cave, Tanintharyi **C, D***Oophanaobtusus***C** specimen NHMUK 1888.12.4.788–790 from Chauktalon, Moulmein with apertural dentition and **D** specimen NHMUK 1871.9.23.64 from Moulmein **E***Oophanalaevis*, possible syntype NHMUK 1899.7.4.11 from Burma.

***Possible syntype***NHMUK ex. Cuming collection (3 shells; Fig. 7A) from Camboja [Cambodia]. NHMUK ex. Cuming collection (2 shells) from Siam. Phra (Buddha) Cave, Tanintharyi Region, Myanmar (11°14'01.5"N, 99°10'42.8"E): CUMZ 13005 (1 shell; Fig. 7B).

##### Diagnosis.

*Oophanamouhoti* can be distinguished from *O.obtusus* (Stoliczka, 1871) by its higher spire, expanded aperture lip, deflected last whorl, and slightly angulate penultimate whorl. In contrast, *O.obtusus* exhibits a narrow aperture, spire convex, periphery of penultimate whorl usually equal to last whorl, strong columellar lamella, and small tubercle near the posterior angle of aperture.

##### Description.

Shell oblique-ovate, white, and translucent; whorls 6½; spire elevated conical with distinct suture. Shell surface glossy with fine transverse ridges that diminish below the periphery; following whorls regularly coiled. Shell periphery rounded and last whorl axially deflected. Aperture subcircular; peristome expanded, thickened, discontinuous, and reflected. Apertural dentition with one strong parietal, one small upper palatal, one small palatal, and one basal lamella. Umbilicus open and deep (Fig. 7A, B).

##### Distribution.

This species has been recorded from several localities in Peninsular Thailand (Siriboon et al. 2020) along the southeastern part of the Tenasserim Range. However, only one specimen was collected in Myanmar from the Tanintharyi Region.

##### Remarks.

The only shell was collected from the limestone hills at Buddha Cave, Tanintharyi Region; it matches well with this species.

*Oophanamouhoti* was originally described based on specimens in the collection of H. Cuming with the very brief collection locality given as Siam. The NHM, London holds a lot of three specimens in the Cuming collection collected by H. Mouhot but has Camboja as the collection locality. This specimen lot does not have Pfeiffer’ handwriting on the label; therefore, they are considered possible syntypes. However, Mouhot’s recorded localities were generally imprecise. No additional specimens for this species are available, nor are records or literature references from Cambodia, other than peninsular Thailand (Sutcharit et al. 2020). A similar situation of imprecise type locality records with Mouhot-collected specimens have been clarified in the case of helicarionid species (see Pholyotha et al. 2020). The recent works on land snails from Thailand confirmed that the specimens from the limestone hills along peninsular Thailand identified as *O.mouhoti* match well with the possible syntype. Therefore, peninsular Thailand (Petchaburi and Prachuap Khiri Khan provinces) are possibly the collecting locality for this species. Furthermore, H. Mouhot visited Petchaburi Province once from May to August in 1860 [see travel routes map in Mouhot (1864a, b)]. Additionally, *Streptaxisjohswichi* Martens, 1864 was described based on specimens collected from Petchaburi Province, Thailand. Later, Martens (1867) examined the *O.mouhoti* specimens in the Cuming collection and agreed that they belonged to the same species.

#### ﻿ Oophana obtusus

Taxon classificationAnimaliaStylommatophoraStreptaxidae

(Stoliczka, 1871)

DCD681A0-8355-5FE9-BCD3-3D37C19E8C1C

Fig. 7C, D

Streptaxis obtusus Stoliczka, 1871: 166, 167, pl. 7, figs 11–13, pl. 8, figs 1–4. Type locality: Prope Moulmein, provincia Tenasserim [Mawlamyine District, Mon State, Myanmar]. Pfeiffer 1876: 495. Nevill 1878: 3. Tryon 1885: 76, pl. 15, fig. 45. Gude 1903: 323, pl. 12, figs 8–10. Blanford and Godwin-Austin 1908: 9.
Odontartemon (Oophana) obtusus – Kobelt 1906: 106, pl. 56, figs 1–3.
Oophana obtusa – Richardson 1988: 236.

##### Material examined.

Moulmein: NHMUK 1903.7.1.4002 (2 shells) ex. Godwin-Austen collection. NHMUK 1871.9.23.64 (1 shell, Fig. 7D). NHMUK 1937.7.91–92. Chauktalon, Moulmein: NHMUK 1906.2.2.342 (3 shells + 1 juvenile) ex. Blanford collection. NHMUK 1888.12.4.788–790 (3 shells, Fig. 7C) ex. Blanford collection. Sacred Lakes, Moulmein: NHMUK 1889.3.15.1 (1 shell) ex. Godwin-Austen collection.

##### Description.

Shell oblique-ovate, white, translucent; whorls 6–7; spire convex with distinct suture. Shell surface glossy with fine transverse ridges, nearly smooth with few transverse ridges near peristome. Embryonic shell ~ 2½ whorls with smooth surface; following whorls regularly coiled. Shell periphery rounded; last whorl slightly axially deflected. Aperture subcircular; peristome continuous, expanded, slightly reflected, and very short sinulus. Apertural dentition with one strong parietal, one palatal, and one strong columella lamella. Umbilicus open and deep (Fig. 7C, D).

##### Distribution.

This species is only known from the type locality, the southern part of Myanmar (Stoliczka 1871).

##### Remarks.

No new specimens were collected in this survey; however, authenticated museum specimens were examined. Regarding genitalia, *Oophanaobtusus* and *Haploptychiusburmanicus* were the first two species anatomically examined and illustrated among several streptaxids in Myanmar by Stoliczka (1871), who noted that there was no noticeable difference in reproductive forms between these two species. Based on the genitalia drawing, *O.obtusus* has a thick penial sheath covering almost the entire penis, and vas deferens not inserted into the penial sheath, but the lengths of vagina and atrium are unclear. Thus, the description and drawing remain insufficient to confirm the distinguishing characters of this species.

#### ﻿ Oophana laevis

Taxon classificationAnimaliaStylommatophoraStreptaxidae

(Blanford, 1899)

853E2DEF-00DF-532F-88CA-302F5F4ECDE5

Fig. 7E

Streptaxis laevis Blanford, 1899: 765, pl. 50, figs 11–12. Type locality: Tenasserim [probably Tanintharyi Region, Myanmar]. Gude 1903: 218. Blanford and Godwin-Austen 1908: 13.
Streptaxis (Odontartemon) laevis – Kobelt 1905: 93, 94, pl. 60, figs 11, 12.
Odontartemon (Oophana) laevis – Kobelt 1910: 150.
Indoartemon laevis – Richardson 1988: 225. Siriboon et al. 2014b: 163.

##### Material examined.

***Possible syntype***NHMUK 1899.7.4.11 (1 shell; Fig. 7E) ex. Beddome collection from Burma [Myanmar].

##### Diagnosis.

*Oophanalaevis* differs from *O.mouhoti* and *O.obtusus* by having a rounded penultimate whorl, convex spire, and apertural dentition with parietal and basal lamellae. Although *O.mouhoti* and *O.obtusus* have a slightly angular penultimate whorl and elevated spire, *O.mouhoti* usually has only a parietal lamella (sometimes with basal lamella). In contrast, *O.obtusus* has parietal, palatal, and columellar lamellae.

##### Description.

Shell oblique-heliciform, white, and translucent; whorls 5½; spire low convex with distinct suture. Shell surface glossy with transverse ridges that diminish below periphery. Embryonic shell ~ 2½ whorls with smooth surface; following whorls regularly coiled. Shell periphery rounded; last whorl axially deflected. Aperture subquadrangular with sinulus; peristome discontinuous, thin, expanded and reflected. Apertural dentition with one parietal and one basal lamella, and sometimes columellar lamella is absent. Umbilicus open and shallow (Fig. 7E).

##### Distribution.

This species is known only from the type locality, which was mentioned only as ‘Tenasserim’ (Blandford 1899). This locality possibly refers to the Tanintharyi Region, southeastern Myanmar.

##### Remarks.

No new specimens were collected in this study. However, we compared the possible type specimen with the other congeners. Blanford (1899) clearly stated that the type series was collected by Beddome and consisted of three specimens. However, the NHM, London holds a lot of only a single specimen from the Beddome collection, with the word India, probably added at a later date, on the label. Therefore, we consider this lot to be a possible syntype.

#### ﻿Genus Perrottetia

Taxon classificationAnimaliaStylommatophoraStreptaxidae

Kobelt, 1905

9A392786-DE75-5F40-9485-9C8E103277A1

Odontartemon (Perrottetia) Kobelt, 1905: 91. Kobelt 1906: 108. Thiele 1931: 730. Forcart 1946: 215.
Oophana (Perrottetia) – van Benthem Jutting 1954: 95.
Perrottetia – Zilch 1960: 562, 563. Richardson 1988: 237. Schileyko 2000: 777, 778. Siriboon et al. 2013: 44, 45.

##### Type species.

*Helixperrotteti* Petit, 1841 by subsequent designation by Forcart (1946: 215).

##### Diagnosis.

Shell oblique-heliciform, and translucent. Longitudinal furrows present behind apertural lip. Apertural dentition usually consists of two parietal, one palatal, one basal and one columellar lamella, and upper palatal and supracolumellar lamellae may be present. Genitalia with long and slender penis, penial sheath thin and not extending the entire penis length. Penial hooks dense, pale brown, expanded at base, with pointed tips, located on penial papillae, and curved towards genital orifice. Vaginal hooks may be present.

##### Remarks.

*Perrotettia* and *Discartemon* are generally similar in having complex apertural dentition; however, their shell morphology is obviously different. *Perrottetia* has a sub-heliciform shell, smaller size, last whorl rounded and more or less deflected, and mostly with two parietal lamellae. Although parietal, palatal, basal and columellar lamellae are always present in *Perrottetia*, possession of second parietal, upper palatal and supracolumellar lamellae is variable, as is the presence of bifid lamellae (Siriboon et al. 2013; Inkhavilay et al. 2016; Bui et al. 2019). In contrast, *Discartemon* exhibits flattened to globose-heliciform shells, last whorl rounded to angular and less deflected with, usually, one parietal lamella.

Nonetheless, some *Discartemon* species from Thailand have two parietal lamellae, for example, *D.afthonodontia* (see Siriboon et al. 2014a: fig. 7a, b) and *D.flavacandida* (see Siriboon et al. 2014a: fig. 7e, f). Likewise, some *Perrottetia* species are also described with only a single parietal lamella, for example, the Vietnamese species *P.aberrata* (Souleyet, 1852) and *P.namdongensis* Bui & Do, 2019 (see Bui et al. 2019: figs 2c–e, 3a–c), and the Laos species *P.unidentata* Inkhavilay & Panha, 2016 (see Inkhavilay et al. 2016: fig. 5f–i).

At present, the genus *Perrottetia* is comprised of about 30 nominal species distributed from India and Sri Lanka to Indochina and southern China (Kobelt 1906; Richardson 1988; Schileyko 2011; Siriboon et al. 2013; Inkhavilay et al. 2016). So far, *P.theobaldi* Benson, 1859 is the only species known from Myanmar.

#### ﻿ Perrottetia theobaldi

Taxon classificationAnimaliaStylommatophoraStreptaxidae

(Benson, 1859)

B211E5E3-5F9E-5D55-AC00-6061BE7488BD

Fig. 8

Streptaxis theobaldi Benson, 1859b: 187. Type locality: Nauclai (lat. 25°15', long. 92°30') [probably in Meghalaya State, India]. Pfeiffer 1868: 449. Hanley and Theobald 1870: 4, pl. 8, fig. 9. Nevill 1878: 3. Tryon 1885: 77, pl. 16, figs 14, 86. Blanford and Godwin-Austen 1908: 9, 10. Ramakrishna et al. 2010: 189.
Odontartemon (Perrottetia) theobaldi –Kobelt 1906: 111, 112, pl. 57, fig. 25. Kobelt 1910: 151.
Perrottetia theobaldi – Richardson 1988: 242, 243. Gittenberger et al. 2021: 74, fig. 1.

##### Material examined.

**Figure 8.**
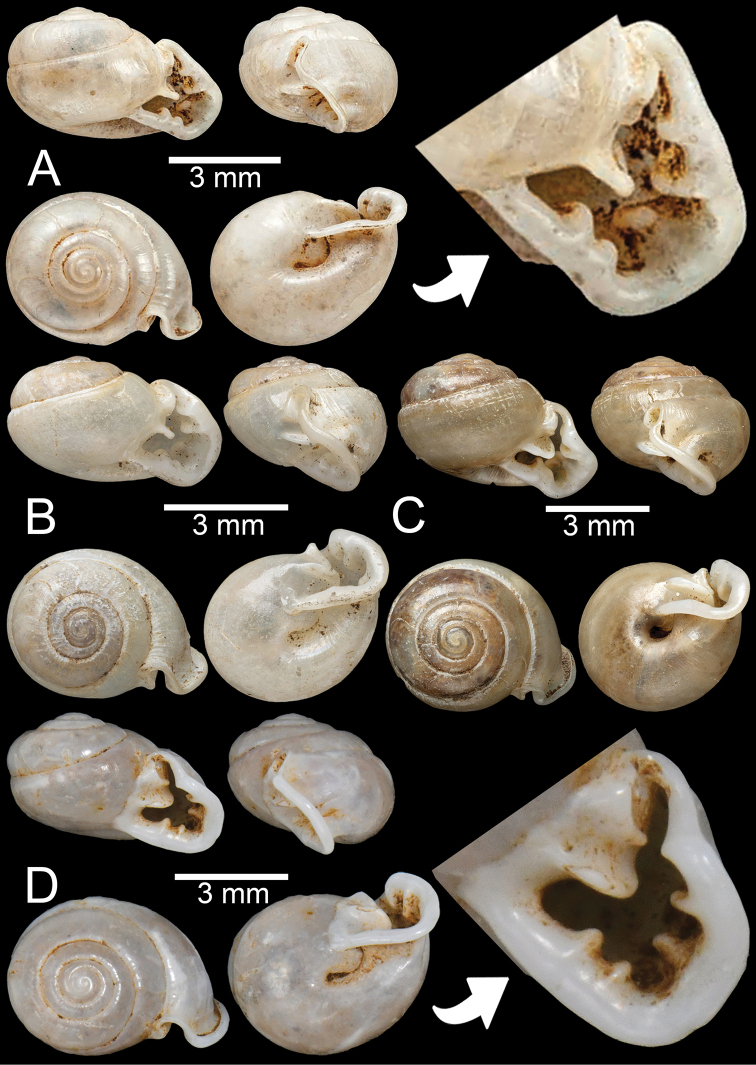
*Perrottetiatheobaldi***A** syntype UMZC I.102535 (no locality data) with apertural dentition **B** specimen NHMUK 1871.9.23.143 from Burma **C** specimen NHMUK 1903.7.1.4005 from Siam boundary **D** specimen CUMZ 13006 from Aik Kham Cave, Taunggyi, Shan State with apertural dentition.

***Syntype*** UMZC I.102535 (2 shells) from unknown locality. Khasi Hill: NHMUK 1888.12.4.784–786 (3 shells; Fig. 8A) ex. Theobald collection. Siam boundary: NHMUK 1903.7.1.4005 (7 shells + 1 juvenile; Fig. 8C) ex. Godwin-Austen collection. Burma: NHMUK 1871.9.23.143 (1 shell; Fig. 8B). Aik Kham Cave, Taunggyi Township, Shan State, Myanmar (20°49'07.0"N, 97°13'42.0"E): CUMZ 13006 (4 shells; Fig. 8D).

##### Diagnosis.

*Perrottetiatheobaldi* is similar to *P.dugasti* (Morlet, 1892) from Vietnam; however, the former species can be distinguished by having a second parietal lamella running to the main parietal lamella, thicker and less reflected peristome, subquadrangular aperture, and shallow suture. In contrast, *P.dugasti* has a weak second parietal lamella running to the sinulus, thinner and more reflected peristome, semi-ovate aperture, and a deeper suture with a clear bifid columellar lamella. Similarly, *P.mabillei* (Bavay & Dautzenberg, 1903) from Vietnam can be separated from *P.theobaldi* by its strong radial ridges, wide and short sinulus, basal lamella absent, bifid columellar lamellae, and deep umbilicus, while *P.theobaldi* has a smooth shell surface, long and narrow sinulus, strong basal lamella, columellar lamellae separated, and shallow umbilicus. Furthermore, *P.theobaldi* differs from the Thai species *P.aquilonaria* Siriboon & Panha, 2013 by having a shallow suture, narrow and long sinulus, rounded last whorl, peristome thicker and less reflected, aperture subquadrangular and columellar lamellae are separated. In contrast, *P.aquilonaria* possesses deep suture, shorter and wider sinulus, the last whorl shouldered, aperture subcircular, more expanded peristome, and bifid columellar lamella.

##### Description.

Shell sub-oblique heliciform, white, and translucent; whorls 5–5½; spire convex with distinct suture. Shell surface glossy with transverse ridges that diminish below periphery. Embryonic shell ~ 2½ whorls with smooth surface; following whorls regularly coiled. Shell periphery rounded; last whorl axially deflected; two deep and short longitudinal furrows present. Aperture triangular with sinulus; peristome continuous, thickened, expanded, and reflected. Apertural dentition with one large and strong parietal, nearly adjoined small second parietal lamella; adjoined at right angles, one small upper palatal, one strong palatal, one strong basal, and bifid columellar lamella. Columellar lamella is sometimes absent. Umbilicus narrow (Fig. 8).

##### Distribution.

This species has been widely recorded from India to Bhutan and Myanmar (Blanford and Godwin-Austen 1908; Ramakrishna et al. 2010; Gittenberger et al. 2021). In Myanmar, the previous record was from Bhamo, Kachin State (Blanford and Godwin-Austin 1908) and the empty shells together with *H.thebawi* (Godwin-Austin, 1888) were found from Shan State in this sampling.

##### Remarks.

All the specimens of *P.theobaldi* (Fig. 8) showed slight differences in the apertural dentition and peristome positions, particularly the columellar and supracolumellar lamellae that are closer or almost united compared to the specimens from the Aik Kham Cave, Shan State (Fig. 8D). However, all were collected from the same range; we place them as the same species.

#### ﻿Genus Haploptychius

Taxon classificationAnimaliaStylommatophoraStreptaxidae

von Möllendorff, 1906

99F5972C-942A-5212-9CED-D6048DF5B84E

Haploptychius von Möllendorff in Kobelt, 1906: 127. Zilch 1960: 562. Richardson 1988: 211. Schileyko 2000: 796, 797. Inkhavilay et al. 2016: 26, 27. Do et al. 2015: 40. Bui et al. 2019: 90.
Odontartemon (Haploptychius) – Thiele 1931: 730. Forcart 1946: 215.
Oophana (Haploptychius) – van Benthem Jutting 1954: 76, 95.

##### Type species.

*Streptaxissinensis* Gould, 1859, by original designation.

##### Diagnosis.

Shell oblique-heliciform to ovate and last whorl deflected. Penultimate whorl round to bluntly angular and extended beyond last whorl. Apertural dentition includes only one parietal lamella. Genitalia with penial sheath thin to thick and extending ~ 1/2 to entire penis length. Penial hooks dense, slightly expanded at base, tips pointed, located on low to high conical papillae and vaginal hooks absent.

##### Remarks.

*Haploptychius* is almost identical with *Carinartemis* in having a deflected last whorl, extended penultimate whorl, and bearing a single parietal lamella. However, *Haploptychius* can be recognized by its oblique-ovate shell and rounded to angular penultimate whorl, while *Carinartemis* possesses an oblique-heliciform shell with sharply keeled penultimate whorl. In genitalia, *Haploptychius* has long and slender penial hooks, without vaginal hooks, while *Carinartemis* possesses shorter and blunt penial hooks, and sometimes transparent vaginal hooks may be present (Siriboon et al. 2014b; Inkhavilay et al. 2016).

A recent molecular phylogeny revealed that *Haploptychius* is polyphyletic, and the traditional genus concept using shell shape and apertural dentition seems unreliable (Siriboon et al. 2020). However, further intensive genital examination and molecular analysis of the type species are necessary to restrict the *Haploptychius* s.s., and generic revision absolutely requires both forms of evidence. Therefore, since the morphological and molecular evidence have never been evaluated, we follow the traditional genus concept in recognizing the streptaxids with oblique-heliciform shells and only one parietal lamella as members of the genus *Haploptychius* s.l.

Currently, the genus *Haploptychius* is comprised of about 40 nominal species distributed from India to Indochina, southern and central China, and Sulawesi of Indonesia (Kobelt 1906; Blanford and Godwin-Austen 1908; Zilch 1961; Richardson 1988; Schileyko 2000; Do and Do 2015; Inkhavilay et al. 2016). In addition, five species of *Haploptychius* s.l. have been documented mainly in southeastern Myanmar (Blanford and Godwin-Austen 1908), and three new species are added in this study.

#### ﻿ Haploptychius bombax

Taxon classificationAnimaliaStylommatophoraStreptaxidae

(Benson, 1859)

2913BF32-586B-5056-921F-CDD085145DA2

Fig. 9

Helix bombax Benson, 1859b: 186. Type locality: Moulmein, necnon and Phie Than in provincia Tenasserim [Mawlamyine, Mon State and Payathonzu, Kayin State, Myanmar]. Pfeiffer 1868: 151. Hanley and Theobald 1870: 15, pl. 31, figs 1, 4.
Streptaxis bombax – Stoliczka 1871: 167. Hanley and Theobald 1870: 63, pl. 156, fig. 9. Nevill 1878: 3. Blanford and Godwin-Austen 1908: 4, 5.
Haploptychius bombax – Kobelt 1906: 147, 148, pl. 63, figs 7–9. Richardson 1988: 212.

##### Material examined.

**Figure 9.**
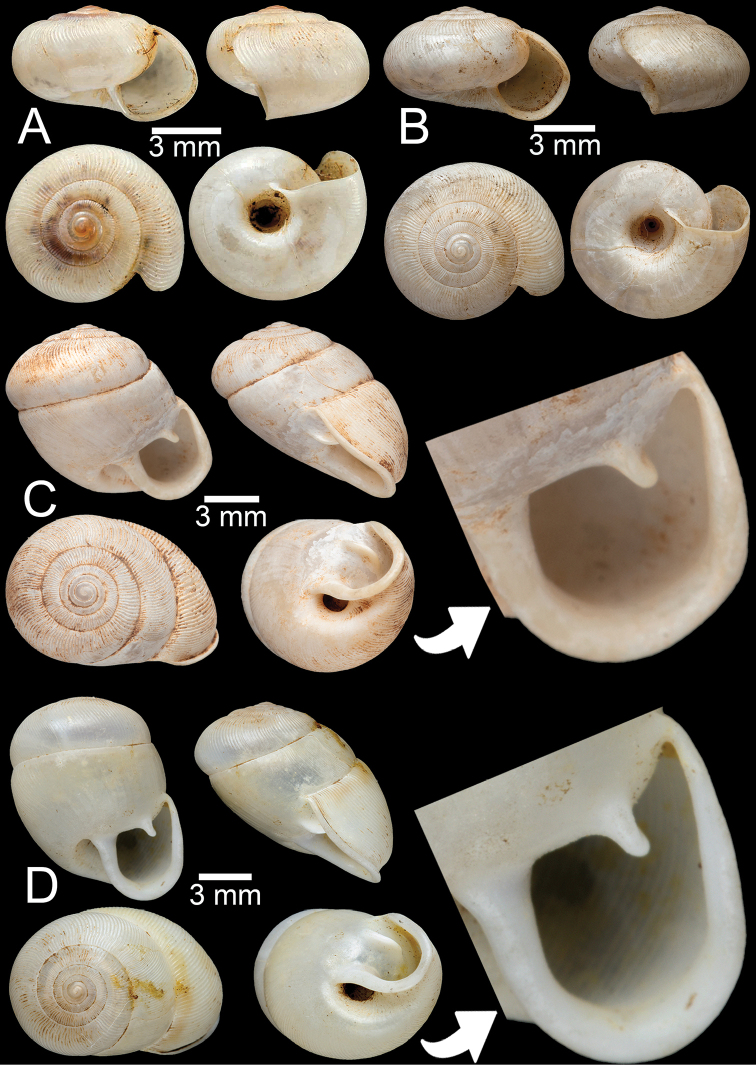
*Haploptychiusbombax***A** syntype UMZC I.102470 from Moulmein **B** juvenile specimen NHMUK 1906.2.2.159 from Moulmein **C** specimen NHMUK 1903.7.1.3990 from Moulmein with apertural dentition **D** specimen NHMUK 1888.12.4.768–770 from Moulmein with apertural dentition.

***Syntype*** UMZC I.102470 (3 shells; Fig. 9A) from Moulmein. India [error]: NHMUK ex. Cuming collection (1 juvenile). Moulmein: NHMUK 1903.7.1.3990 (4 shells; Fig. 9D) ex. Godwin-Austen collection. NHMUK 1906.2.2.159 (1 juvenile; Fig. 9B) ex. Blanford collection. NHMUK 1906.2.2.340 (2 shells) ex. Blanford collection. NHMUK ex Godwin-Austen collection (2 shells). NHMUK 1888.12.4.768–770 (3 shells; Fig. 9C).

##### Diagnosis.

*Haploptychiusbombax* can be differentiated from *H.burmanicus* by having a large oblique-ovate shell, without sinulus, and larger or wider last whorl. In contrast, *H.burmanicus* possesses a small sub-oblique heliciform shell, wide sinulus, and compressed last whorl. Compared with *H.pellucens* (Pfeiffer, 1863) from Laos, *H.bombax* differs by having strong radial ridges, convex spire, and narrow umbilicus, while *H.pellucens* exhibits a nearly smooth surface, more ovate shape, elevated spire, and umbilicus widely open.

##### Description.

Shell oblique-ovate, white, and translucent; whorls 5½–7; spire convex with distinct suture. Shell surface glossy with fine transverse ridges that diminish below periphery of penultimate whorl. Embryonic shell ~ 2½ whorls with smooth surface; following whorls regularly coiled. Shell periphery rounded; last whorl axially deflected. Aperture semi-ovate; peristome discontinuous, thickened, slightly expanded, and reflected. Apertural dentition with one strong parietal lamella. Umbilicus open and deep (Fig. 9).

##### Distribution.

*Haploptychiusbombax* is only known from the type locality in southeastern Myanmar (Benson 1859b).

##### Remarks.

The original description mentioned only 5½ whorls, but the museum specimen (Fig. 9C, D) had 7 whorls. This study collected no specimens, but authenticated museum specimens (juveniles and adults) were examined.

#### ﻿ Haploptychius blanfordi

Taxon classificationAnimaliaStylommatophoraStreptaxidae

(Theobald, 1864)

E0ADEBC8-7C2B-57F5-8170-10371DA2C99E

Fig. 10A–C

Streptaxis blanfordi Theobald, 1864: 245. Type locality: Montibus Arakanensibus provincia Pegu [Arakan Mountains in Bago Region, Myanmar]. Nevill 1878: 2.
Streptaxis birmanica Blanford, (in MSS.) Theobald, 1864: 245, 246 (in part). Type locality: Pegu [Bago Region, Myanmar]. Theobald 1865: 277.
Streptaxis blanfordianus Stoliczka 1871: 163, pl. 7, figs 8, 9 (unjustified emendation). Pfeiffer 1876: 494.
Streptaxis birmanica – Hanley and Theobald 1876: 4, pl. 8, fig. 5 (non Theobald 1864).
Streptaxis blanfordi var. – Godwin-Austen 1895: 443.
Haploptychius blanfordianus – Kobelt 1906: 143, 144, pl. 57, fig. 15, pl. 62, figs 4, 5.
Haploptychius blanfordi – Richardson 1988: 212.
Streptaxis blanfordi – Hanley and Theobald 1870: 4, pl. 8, fig. 10 (non Theobald 1864).
Streptaxis birmanicus [sic] – Richardson 1988: 251.

##### Material examined.

**Figure 10.**
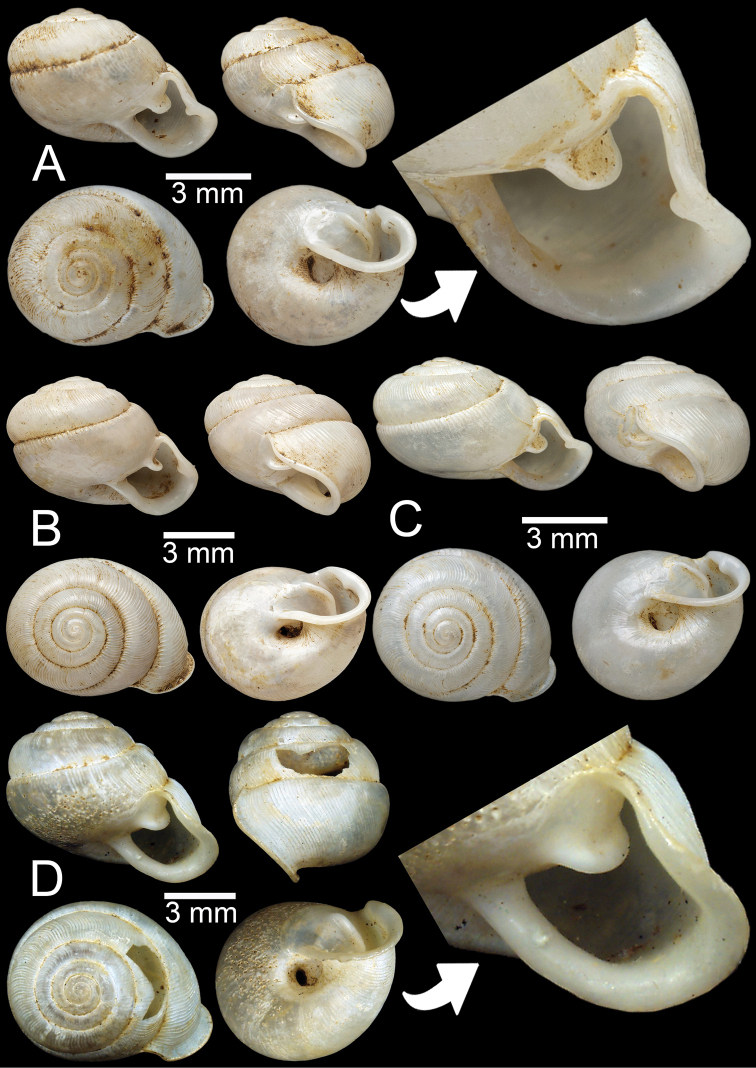
**A–C***Haploptychiusblanfordi***A** syntype NHMUK 1888.12.4.777–779 from Pegu with apertural dentition **B** specimen NHMUK 1906.2.2.338 from Akouktoung, Pegu **C** specimen NHMUK 1909.3.15.71 from Pegu **D***Haploptychiusburmanicus*, syntype NHMUK 1906.2.2.199 from Tongoop, Arakan with apertural dentition.

***Syntype***NHMUK 1888.12.4.777–779 (3 shells; Fig. 10A) from Pegu.NHMUK 1909.3.15.71 (1 shell; Fig. 10C) ex. Godwin-Austen collection. Pegu, Arakan: NHMUK 1909.3.15.93 (3 shells) ex. H. Blanford collection. Akouktoung, Pegu: NHMUK 1906.2.2.338 (4 shells; Fig. 10B) ex. W.T. Blanford collection. Pegu, Arakan: NHMUK 1909.3.15.93 (3 shells) ex. H. Blanford collection.

##### Diagnosis.

*Haploptychiusfischeri* (Morlet, 1887) almost shares the same shell form as *H.blanfordi*, but the latter possesses a more depressed shell, penultimate whorl more bulging, aperture wider, longer and subquadrangular shape.

##### Description.

Shell oblique-heliciform, white, and translucent; whorls 6; spire convex with distinct suture. Shell surface glossy with fine transverse ridges that diminish below periphery. Embryonic shell ~ 2½ whorls with smooth surface; following whorls regularly coiled. Shell periphery rounded; last whorl axially deflected. Aperture semi-ovate; peristome discontinuous, thickened, expanded, and reflected. Apertural dentition with one strong parietal and small palatal lamellae. Umbilicus open and deep (Fig. 10A–C).

##### Distribution.

This species has been recorded with a wide distribution range from Arakan [Rakhine State], Pegu [Bago Region], and Shan State in Myanmar. However, the record from Cocos Islands in the southern Indian Ocean by Blanford and Godwin-Austen (1908) needs to be verified with modern evidence rather than by shell morphology alone.

##### Remarks.

The taxonomic status of this species is still ambiguous because it is identical in shell morphology to *Streptaxisbirmanica*. Theobald (1864) consecutively described *Streptaxisblanfordi* and *Streptaxisbirmanica* based on specimens from the same geographical area as Pegu. These two nominal species resemble each other in having one parietal and one palatal lamella, whereas they differ in only minor characters of shell shape and size. In this revision, we consider these two nominal species as synonyms. Only the type specimens of *H.blanfordi* could be located and examined; we therefore consider *Streptaxisbirmanica* as the junior synonym.

The original spelling of the species was *blanfordi*, which was intentionally modified to *blanfordianus* by Stoliczka (1871) but for an unclear reason. This subsequent spelling of *blanfordianus* is an unjustified emendation made available with its own authorship and date, and so became a junior objective synonym (ICZN 1999: Arts 32.3, 33.2.3, 50.5).

Theobald (1864) clearly stated that *Streptaxisbirmanica* W. Blanford, (in MSS.) was described based on a single specimen received from W.T. Blanford. However, we could not locate this single shell as the holotype fixed by monotypy. Theobald (1864) also stated that the other two shells lacked the palatal lamella and were of a relatively smaller size, which are recognized as distinct varietal entities. Therefore, these two specimens are excluded from the type series of *Streptaxisbirmanica*. The authorship was originally attributed to W.T. Blanford as the manuscript name. However, since Blanford did not write the description or credit the description to W.T. Blanford, the taxon is attributed to Theobald only (ICZN 1999: Art. 50.1.1).

#### ﻿ Haploptychius burmanicus

Taxon classificationAnimaliaStylommatophoraStreptaxidae

(Blanford, 1865)

7F898D45-2D17-5A49-97BE-32520F4F8FCB

Fig. 10D

Streptaxis burmanica Blanford 1865: 81, 82. Type locality: Tongoop, Arakan [Toungup, east of Arakan Hills, Thandwe District, Rakhine State, Myanmar].
Streptaxis burmanicus [sic] – Pfeiffer 1868: 444. Stoliczka 1871: 163, pl. 7, figs 5–7. Nevill 1878: 2. Blanford and Godwin-Austen 1908: 6, fig. 5. Richardson 1988: 251.
Haploptychius burmanicus – Kobelt 1906: 145: pl. 57, figs 19, 20, pl. 62, figs 1–3. Richardson 1988: 213. Ramakrishna et al. 2010: 190.

##### Material examined.

***Possible syntype***NHMUK 1906.2.2.199 (2 adults + 1 juvenile; Fig. 10D) ex. W.T. Blanford collection from Tongoop, Arakan [Taungup, Rakhine State].

##### Description.

Shell oblique-heliciform, white, and translucent; whorls 6; spire convex with distinct suture. Shell surface glossy with fine transverse ridges that diminish below periphery. Embryonic shell ~ 2½ whorls with smooth surface; following whorls regularly coiled. Shell periphery rounded; last whorl axially deflected. Aperture semi-ovate; peristome continuous; sometimes discontinuous, thin, expanded, and slightly reflected. Apertural dentition with one strong parietal lamella. Umbilicus open and deep (Fig. 10D).

##### Distribution.

*Haploptychiusburmanicus* is still known only from the type locality in southern Myanmar (Blanford 1865). Outside Myanmar, it is also reported from Mizoram in India (Ramakrishna et al. 2010); however, this record needs to be verified.

##### Remarks.

This species highly resembles *H.blanfordi* in shell form, but *H.burmanicus* is more globosely heliciform, with a higher spire and only one parietal lamella. Without anatomical information, the generic placement of this species is still tentative, and we retain this as Blanford and Godwin-Austen (1908). In addition, *H.perlissus*Vermeulen et al. 2019 from Vietnam (Vermeulen et al. 2019: figs 49–51) can be distinguished from *H.burmanicus* by having an oblique-ovate shell, less extended penultimate whorl from the last whorl, lower to nearly flattened spire, the palatal side is almost straight, basal side rounded, and columellar side broadly rounded, whereas *H.burmanicus* has a higher spire, more compressed shell, and the last whorl is more axially deflected.

#### ﻿ Haploptychius solidulus

Taxon classificationAnimaliaStylommatophoraStreptaxidae

(Stoliczka, 1871)

9E7CAB4E-AA64-58BA-9635-E9AA97037195

Figs 1 , 11

Streptaxis solidulus Stoliczka, 1871: 166, pl. 7, fig. 10. Type locality: Moulmein, Tenasserim [Mawlamyine District, Mon State, Myanmar]. Pfeiffer 1876: 494. Hanley and Theobald 1870: 40, pl. 98, fig. 7. Nevill 1878: 3. Tryon 1885: 71, pl. 14, fig. 99. Gude 1903: 212. Blanford and Godwin-Austen 1908: 7. Kobelt 9010: 152.
Haploptychius solidulus – Richardson 1988: 221, 222.

##### Material examined.

**Figure 11.**
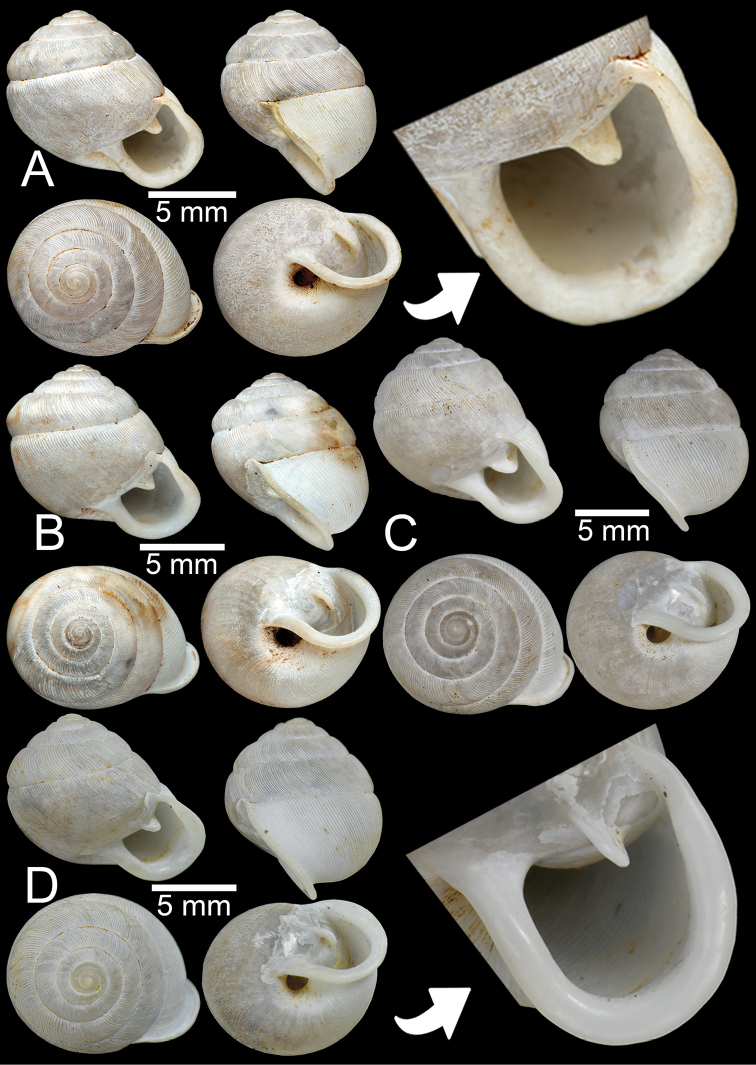
*Haploptychiussolidulus***A** specimen NHMUK 1909.3.15.75 from Moulmein with apertural dentition **B** specimen NHMUK 1888.12.4.774–776 from Molumein **C, D** specimen CUMZ 13007 from Pathen Mountain, Mawlamyine, Mon State with apertural dentition.

Moulmein: NHMUK 1937.7.9.10 (1 shell). NHMUK 1906.2.2.339 (1 shell) ex. Blanford collection. NHMUK 1888.12.4.774–776 (3 shells; Fig. 11B). NHMUK 1909.3.15.75 (1 shell; Fig. 11A) ex. Godwin-Austen collection. Pathen Mountain, east bank of Attaran River and ~ 45 km southeast of Kyaikmaraw Township, Mawlamyine District, Mon State, Myanmar (16°14'7.5"N, 97°56'48.1"E): CUMZ 13007 (15 shells; Fig. 11C, D).

##### Diagnosis.

Among the *Haploptychius* species from Myanmar, this species is distinctly large with an oblique-ovate shell, and less deflected last whorl. *Haploptychiussolidulus* has almost the same shell form as *Oophanamouhoti*. However, it differs from *O.mouhoti* by having somewhat enlarged last whorl, more deflected last whorl, and a conical spire with only one parietal lamella. In contrast, *O.mouhoti* shows a depressed and obtuse conical spire, with one parietal and small basal lamellae.

##### Description.

Shell oblique-ovate, white, and opaque; whorls 6½–7; spire elevated conical with distinct suture. Shell surface glossy with fine transverse ridges that diminish below periphery. Embryonic shell ~ 2½ whorls with smooth surface; following whorls regularly coiled. Shell periphery rounded; last whorl axially deflected. Aperture semi-ovate; peristome discontinuous, thickened, expanded, and slightly reflected. Apertural dentition with one strong parietal lamella. Umbilicus open and deep (Fig. 11).

##### Distribution.

This species appears to be restricted to limestone karsts and is only recorded from southern Myanmar.

##### Remarks.

Stoliczka (1871) mentioned the specimens received from Theobald, who collected at Yethebiankoo [Rathe Pyan Cave] on the Attaran river, south-east of Moulmein [Mawlamyine]. Therefore, the exact type locality is assumed to be Rathe Pyan Cave, but it is located southwest of Hpa-an, Kayin State. Unfortunately, no living specimens could be examined.

#### ﻿ Haploptychius thebawi

Taxon classificationAnimaliaStylommatophoraStreptaxidae

(Godwin-Austin, 1888)

CD2F41B7-771E-529F-8BCA-4B98EF88C87F

Figs 1 , 12

Streptaxis thebawi Godwin-Austin, 1888: 243. Type locality: Pingoung, Shan Hills [Pinlaung Township, Taunggyi District, Shan State, Myanmar]. Gude 1903: 212, 322, 325, pl. 12, figs 11–13.
Haploptychius thebawi – Kobelt 1906: 145, 146: pl. 62, figs 11–13. Richardson 1988: 222.

##### Material examined.

**Figure 12.**
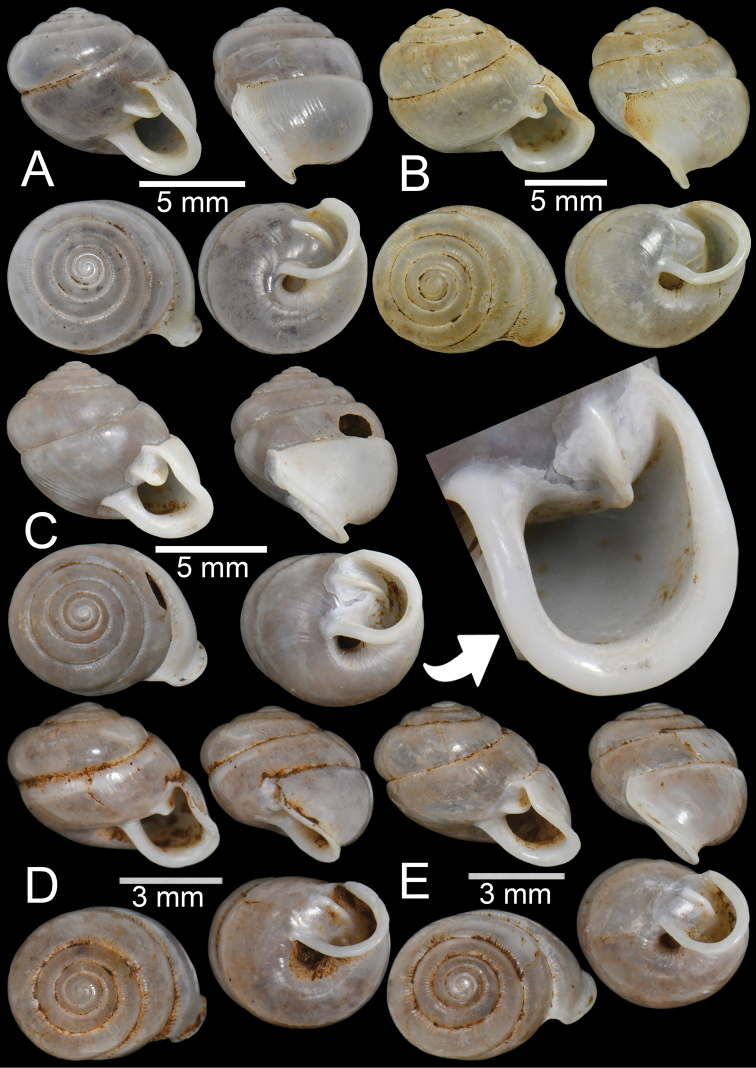
*Haploptychiusthebawi***A–C** specimens CUMZ13008 from Pyinyaung Village, Meiktila, Mandalay Region, with apertural dentition **D, E** specimens CUMZ 13010 from Aik Kham Cave, Taunggyi, Shan State.

Limestone hills (Apache Cement Factory), Pyinyaung Village, Thazi Township, Meiktila District, Mandalay Region, Myanmar (20°49'39.1"N, 96°23'35.1"E): CUMZ 13008 (4 shells; Fig. 12A–C). Ywangan Village, near Lin Way Monastery, Kalaw Township, Taunggyi District, Shan State, Myanmar (21°13'43.3"N, 96°33'19.2"E): CUMZ 13009 (7 shells). Aik Kham Cave, Taunggyi Township, Taunggyi District, Shan State, Myanmar (20°49'07.0"N, 97°13'42.0"E): CUMZ 13010 (3 shells; Fig. 12D, E).

##### Diagnosis.

*Haploptychiusthebawi* is similar to *H.solidulus* in having a high conical spire and globosely ovate shell. The former species can be discriminated by its smoother shell surface and narrower aperture, more rounded and extended penultimate whorl, and less inflated last whorl. Additionally, *H.thebawi* resembles *H.porrectus* (Pfeiffer, 1863) from Laos in having strong radial ridges, conical spire, and an inflated and deflected last whorl, whereas *H.thebawi* exhibits a larger shell, less depressed and deflected last whorl, fine radial ridges, and higher spire.

##### Description.

Shell oblique-heliciform, white and translucent; whorls 5½–6½; spire conical with distinct suture. Shell surface glossy with transverse ridges that diminish below periphery. Embryonic shell ~ 2½ whorls with smooth surface; following whorls regularly coiled. Shell periphery rounded; last whorl axially deflected. Aperture subcircular; peristome discontinuous, thickened, expanded, and slightly reflected. Apertural dentition with one strong parietal lamella. Umbilicus open and deep (Fig. 12).

##### Distribution.

This species occurs in central-eastern Myanmar, based on the type locality and two localities in Shan State, one locality in Mandalay Region, and it is relatively low in population density.

##### Remarks.

Originally, *H.thebawi* was described based on specimens from Pingoung, Shan Hills, which is probably now referred to as Pinlaung Township, Taunggyi District, Shan State. Unfortunately, the type specimens of *H.thebawi* could not be located in the NHM, London collection. However, the specimens examined herein match well with the original description and the syntype illustrated in Gude (1903: figs 11–13). Despite that, the specimens from Mandalay Region tend to have a more ovate shell and elongated last whorl, and their smooth shell surface, narrow umbilicus, and conical spire make them most similar to this nominal species. Therefore, we treat this population as an intraspecific variation. Examination of genitalia of specimens from this population is necessary to clarify their taxonomic status.

Blanford and Godwin-Austen (1908) treated this nominal species as a junior synonym of *H.burmanicus*. However, the recently collected specimens of *H.thebawi* can be distinguished from *H.burmanicus* and *H.blanfordi* by having an oblique-ovate to oblique-heliciform shell, elevated and conical spire, nearly smooth shell surface, penultimate whorl extended beyond last whorl, only one parietal lamella, and narrow umbilicus. Conversely, the two latter species have sub-oblique heliciform shells, depressed spire, prominent radial ridges on the shell surface, penultimate whorl slightly extended beyond the last whorl, with one parietal and one palatal lamella, and a wide umbilicus. Therefore, we consider *H.thebawi* to stand as a separate species.

Among three populations of *H.thebawi* (Table 1), Aik Kham Cave specimens have the smallest size with a more depressed shell, followed by Ywangan Village; Apache Cement Factory specimens have the largest shell with more conical spire and rounded last whorl.

#### ﻿ Haploptychius tenasserimicus

Taxon classificationAnimaliaStylommatophoraStreptaxidae

Man & Panha sp. nov.

F83B2BF8-FE13-5A3E-AA97-39FABE8795F2

https://zoobank.org/2391DEBA-7A84-4520-AF32-7BEB712C45EB

Fig. 13A–C

##### Type material.

**Figure 13.**
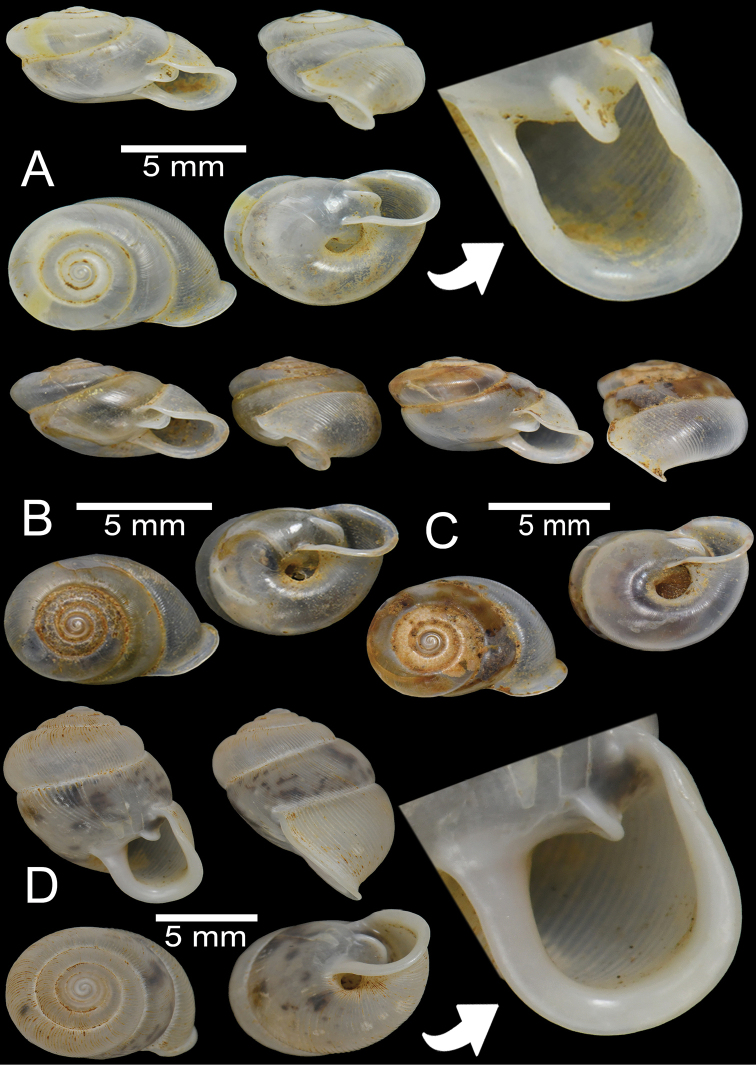
**A–C***Haploptychiustenasserimicus* sp. nov. **A** holotype CUMZ 13011 with apertural dentition **B, C** paratypes CUMZ 13012 from the type locality **D***Haploptychiusheliakosus* sp. nov. holotype CUMZ 13013 with apertural dentition.

***Holotype***CUMZ 13011 (Fig. 13A). Measurements: shell height 4.5 mm, shell width 8.6 mm, and 6 whorls. ***Paratypes***CUMZ 13012 (5 shells; Fig. 13B, C), NHMUK (2 shells).

##### Type locality.

This new species was found from the limestone karsts near Lampane Village, Tanintharyi Region, Myanmar (11°40'18.1"N, 99°13'30.1"E).

##### Etymology.

The specific name *tenasserimicus* refers to the type locality of this new species located on the Tenasserim Mountain Range, which forms the backbone of Indochina.

##### Diagnosis.

This species is distinguishable by its small size, angular penultimate whorl, low convex spire, and aperture that elongates and grows almost horizontally. *Haploptychiustenasserimicus* sp. nov. differs from *H.burmanicus* and *H.blanfordi* species by having oblique-ovate shells, prominent transverse ridges, higher spire, rounded penultimate whorl, and less deflected last whorl. This new species also differs from *H.blaisei* (Dautzenberg & Fischer, 1905) from Laos and Vietnam by having a relatively smaller shell (width ~ 8 mm), nearly flattened spire, angular penultimate whorl and rounded last whorl. In contrast, *H.blaisei* possesses a relatively larger shell (width ~ 10 mm), higher spire, rounded penultimate whorl, and compressed last whorl. In addition, *H.tenasserimicus* sp. nov. can be distinguished from *H.dorri* (Dautzenberg, 1894) from Vietnam (see Inkhavilay et al. 2016) by having a more depressed and larger shell (width ~ 8 mm), angular penultimate whorl, aperture longer and more axially deflected last whorl. *Haplotychiusdorri* exhibits a depressed and smaller shell (width ~ 5 mm), smooth shell surface, rounded penultimate whorl, shorter aperture and last whorl less axially deflected from the vertical axis.

##### Description.

Shell sub-oblique heliciform, white and translucent; whorls 6; spire low convex with distinct suture. Shell surface glossy with fine transverse ridges that diminish below periphery; varices present. Embryonic shell ~ 2½ whorls with smooth surface; following whorls regularly coiled. Shell periphery angular; last whorl axially deflected. Aperture semi-ovate; peristome discontinuous, expanded and slightly reflected. Apertural dentition with one parietal lamella. Umbilicus open and shallow (Fig. 13A–C).

##### Distribution.

This species was only collected from the type locality; limestone hills in primary forest in the Tanintharyi Region, Myanmar.

##### Remarks.

The genitalia information is not known.

#### ﻿ Haploptychius heliakosus

Taxon classificationAnimaliaStylommatophoraStreptaxidae

Man & Panha sp. nov.

F965BA42-B7AA-51CD-9194-423104FA8103

https://zoobank.org/2ED29774-4354-40B7-AC84-8C74CE43AE3E

**Figure 14.**
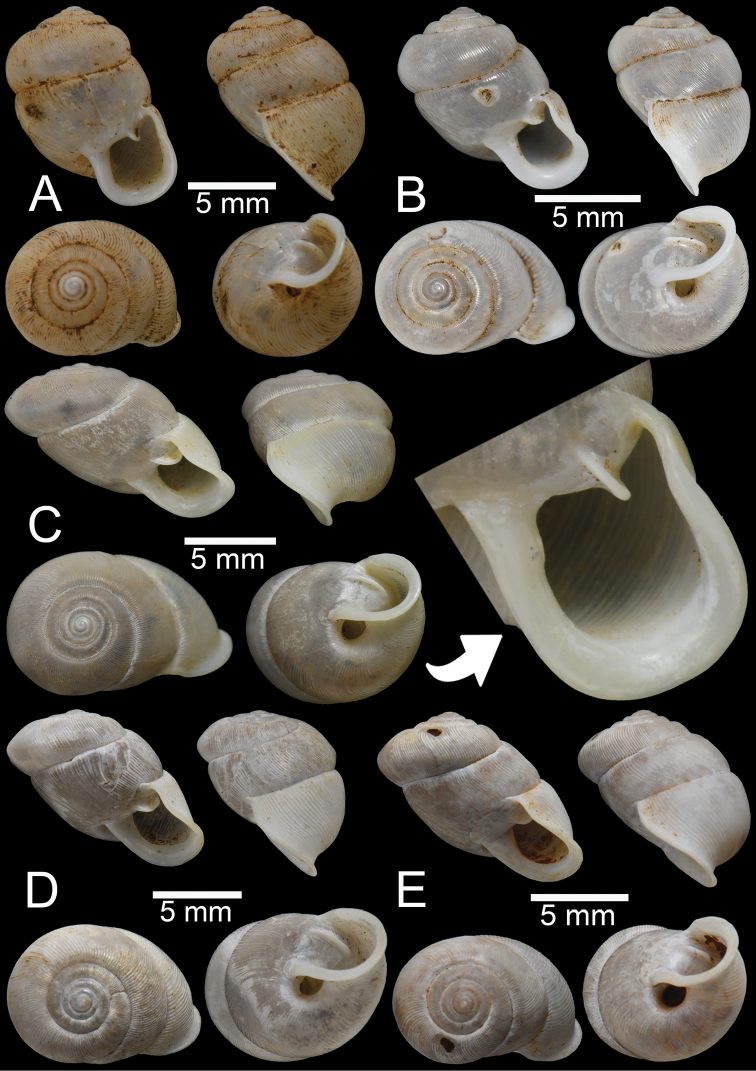
**A, B***Haploptychiusheliakosus* sp. nov. **A** paratype CUMZ 13014 from the type locality and **B** specimen CUMZ 13016 from Kyonknow Cave, Hpa-an, Kayin State **C–E***Haploptychiuskarenorum* sp. nov. **C** holotype CUMZ 13017 with apertural dentition **D** paratype CUMZ 13018 from the type locality and **E** specimen CUMZ 13019 from Taung Lay Cave, Hpa-an, Kayin State.

**Figure 15.**
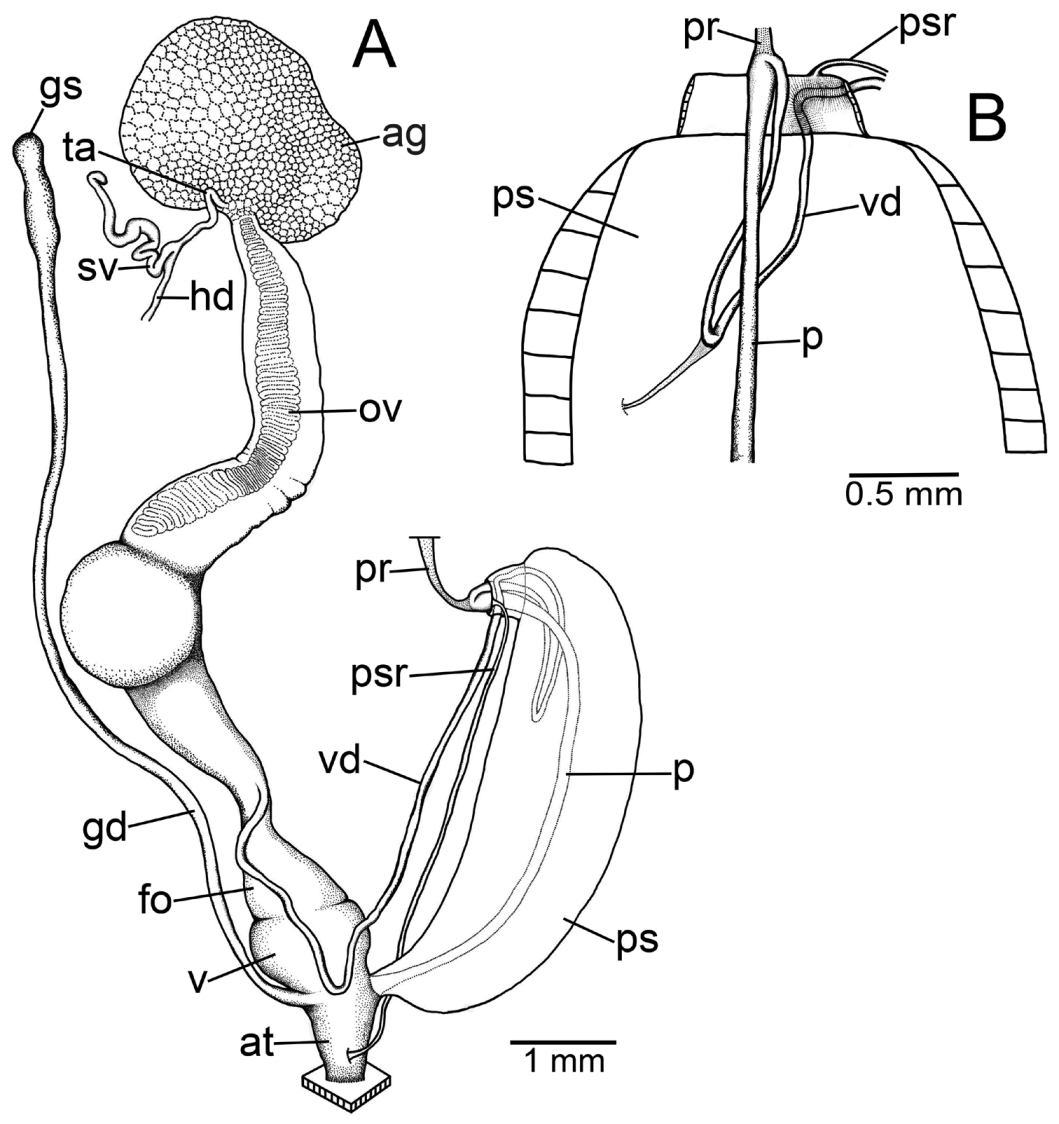
Genital anatomy of *Haploptychiusheliakosus* sp. nov. paratype CUMZ 13014 **A** reproductive system **B** insertion of vas deferens into penial sheath.

**Figure 16.**
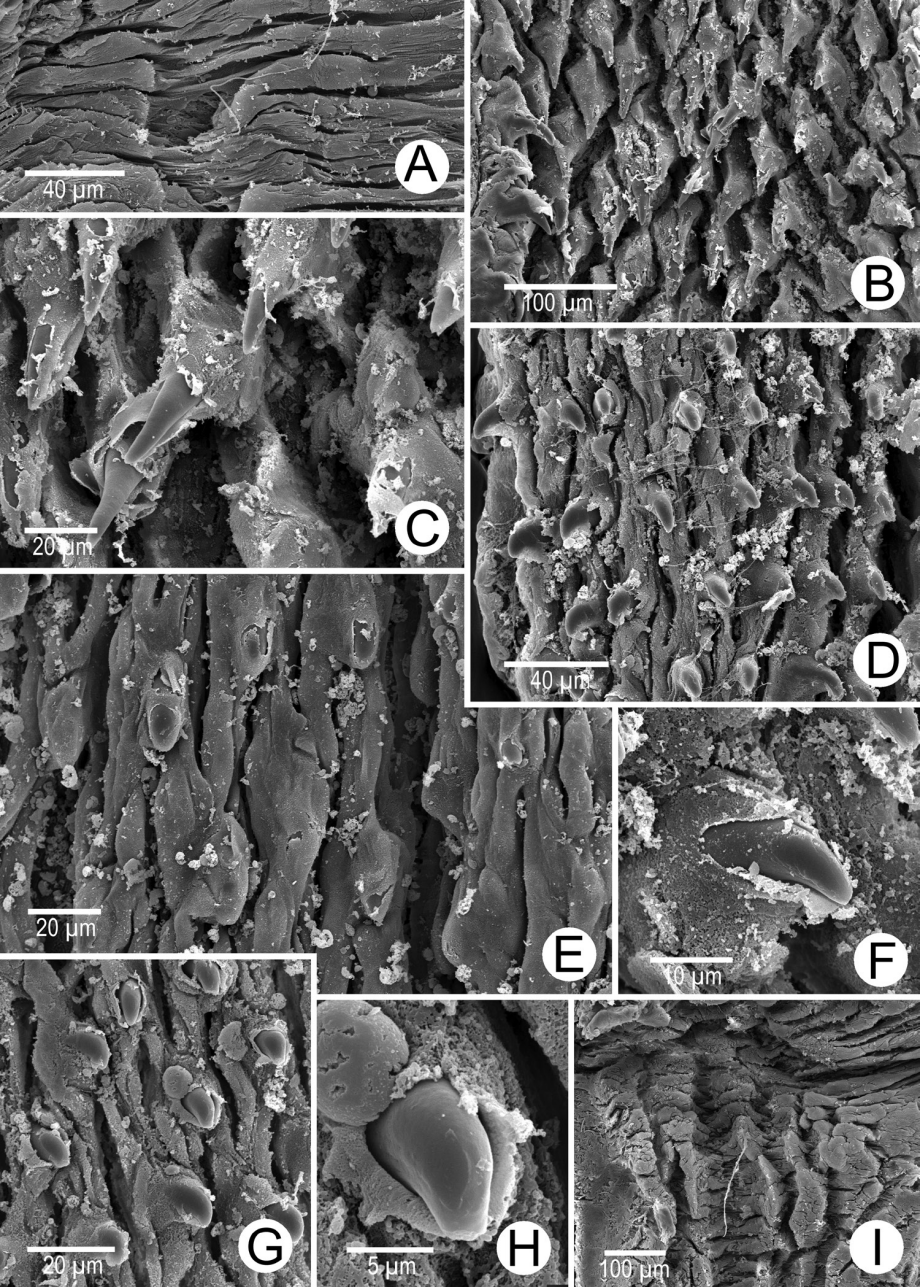
Internal sculpture of genitalia of *Haploptychiusheliakosus* sp. nov. paratype CUMZ 13014 **A** atrium surface **B** arrangement of penial hooks on proximal part of penis **C** top view of penial hook **D, E** arrangement of penial hooks on middle part of penis **F** top view of penial hook with obtuse tip **G** arrangement of penial hooks on distal part of penis **H** high magnification of penial hook with obtuse tip **I** vaginal folds.

Figs 2C , 13D , 14A, B , 15 , 16 , 24C

##### Type material.

***Holotype***CUMZ 13013 (Fig. 13D). Measurements: shell height 9.0 mm, shell width 10.2 mm, and 7 whorls. ***Paratypes***CUMZ 13014 (24 shells; Fig. 14A), CUMZ 13015 (15 specimens in ethanol), NHMUK (2 shells).

##### Type locality.

Bardai Mountain, Hpa-an Township, Hpa-an District, Kayin State, Myanmar (16°59'50"N, 97°41'48"E).

##### Other material examined.

Kyonknow Cave, Hpa-an Township, Hpa-an District, Kayin State, Myanmar (17°01'00.1"N, 97°41'42.1"E): CUMZ 13016 (7 shells; Fig. 14B).

##### Diagnosis.

*Haplotychiusheliakosus* sp. nov. differs from *H.bombax* by having a deeper suture, higher spire, rounded penultimate whorl, and subquadrangular aperture. In contrast, *H.bombax* possesses a relatively shallower suture, lower spire, angular penultimate whorl, and semi-ovate aperture. *Haplotychiusheliakosus* sp. nov. also differs from *H.burmanicus* and *H.blanfordi* by having an oblique ovate shell, higher spire, and less axially deflected last whorl. In contrast, the two latter species exhibit a depressed heliciform shell and lower spire, and more axially deflected last whorl. Although *H.heliakosus* sp. nov. has a shell similar to *H.pellucens* from Laos, this new species has a less axially reflected last whorl, subquadrangular aperture, and more ridges on the shell surface. Additionally, the genitalia of *H.heliakosus* sp. nov. has a thickened penial sheath covering almost the entire penis, and short and stout penial hooks on papillae, while *H.pellucens* has a thin penial sheath covering ~ 1/2 of the penis, and long and slender penial hooks without papillae. This new species differs from *C.exacutus* (Gould, 1856) by having penial sheath retractor muscle originating at atrium, vas deferens passing through a short section of thin penial sheath before extending ~ 1/3 of the penial sheath length to the curved portion, shorter free oviduct, seminal vesicle ca. twice the length from talon to branching point of seminal vesicle, thickened atrial folds with sparse atrial pores, and stout distal penial hooks.

##### Description.

Shell oblique-ovate, white, and translucent; whorls 7–7½; spire low conical with distinct suture. Shell surface glossy with transverse ridge, nearly smooth with few transverse ridges near peristome. Embryonic shell large, ~ 2½ whorls with smooth surface; following whorls regularly coiled. Penultimate whorl rounded; last whorl axially deflected. Aperture subquadrangular; peristome discontinuous, thickened, expanded, and slightly reflected. Apertural dentition with one strong parietal lamella and sometimes with small second parietal lamella adjoined at a right angle. Umbilicus open and deep (Fig. 13D, 14A, B).

***Genital organs*.** Atrium (at) short. Penis (p) very thin, and long tube. Penial sheath (ps) muscularly enlarged, very thickened, and extending entire penis length; penial sheath retractor muscle (psr) thin, originating at atrium, and inserting distally on penial sheath (Fig. 15A). Vas deferens (vd) passes through a short section of thin penial sheath then extends ~ 1/3 of the penial sheath length to a curved portion before entering penis distally. Curved portion with very thin connective tissue attached between vas deferens and penial sheath wall (Fig. 15B). Penial retractor muscle (pr) thin, very long, inserting at junction of penis and vas deferens.

Internal wall of atrium generally smooth with transverse thickened atrial folds with sparse atrial pores (Fig. 16A). Proximal penial wall with scattered pale brownish penial hooks, ~ 26 hooks/200 μm^2^; hooks located on irregular trapezoidal penial papillae and separated by longitudinal folds (Fig. 16B). Penial hooks small (< 0.04 mm in length), slightly expanded at base, tips pointed, and slightly curving away from genital orifice (Fig. 16C). Penial wall on middle to distal parts with scattered light brownish penial hooks, ~ 24 hooks/200 μm^2^; hooks located on laterally-flattened penial papillae separated by longitudinal folds (Fig. 16D, E). Penial hooks short, stout, small (< 0.01 mm in length), expanded at base, tips obtuse and curved towards genital orifice (Fig. 16F–H).

Internal wall of atrium generally smooth with sparse atrial pores (Fig. 23A). Proximal penial wall covered with scattered and pale brownish penial hooks, ~ 12 hooks/200 μm^2^. Proximal penial hooks located on laterally flattened penial papillae; hooks small and short (< 0.03 mm in length), slightly expanded at base, tips obtuse and curved towards genital orifice (Fig. 23B, C). Middle and distal penial walls densely covered with pale brownish hooks, ~ 20 hooks/200 μm^2^. Middle and distal hooks located on laterally compressed penial papillae separated by reticulated folds; hooks small, short (< 0.01 mm in length), slightly expanded at base, tips pointed (Fig. 23D–G).

Vagina very short, stout, and ~ 1/10 of penis length. Gametolytic duct (gd) a long tube extending as far as albumin gland; gametolytic sac (gs) ovate. Proximal free oviduct (fo) enlarged, tapering to a smaller tube in the middle part, then enlarged distally. Oviduct (ov) enlarged and folded; prostate gland inconspicuous and bound to oviduct. Talon (ta) small, short, and club shaped. Hermaphroditic duct (hd) bearing long seminal vesicle (sv) of about twice the length from talon to branching point of seminal vesicle (Fig. 15A).

Vaginal wall with longitudinal vaginal folds (Fig. 16I), folds with nearly smooth surface, and vaginal hook absent.

***Radula*.** Each row consists of ~ 35 teeth with formula (17)–1–(17). Central tooth very small with pointed cusp. Lateral and marginal teeth undifferentiated, lanceolate, unicuspid, and lanceolate. Latero-marginal teeth gradually reduced in size, with outermost teeth much smaller and shorter than inner teeth (Fig. 24C).

##### Etymology.

The specific name *heliakosus* is derived from the Greek word *heliakos* meaning of the sun. It honors our colleague, Dr. Arthit Pholyotha, who collected the specimens and took the photos of the living snails used in this study. His first name Arthit means the Sun.

##### Distribution.

This new species is currently known from two localities in the limestone karsts near Salween (Thanlwin) Basin, Kayin State, southeastern Myanmar.

##### Remarks.

Shell variations of *H.heliakosus* sp. nov. were found between two populations. Specimens from the Kyonknow population (Fig. 14B) have a straighter periphery or nearly cylindrical last whorl compared to those from the type locality, Bardai Mountain (Fig. 13D, 14A). As no living specimens from the Kyonknow Cave were collected, we considered the Kyonknow population as an intraspecific variation of *H.heliakosus* sp. nov. because this locality is very close to the type locality. Living specimens from the Kyonknow population and genital examination are necessary to resolve these systematic issues.

#### ﻿ Haploptychius karenorum

Taxon classificationAnimaliaStylommatophoraStreptaxidae

Man & Panha sp. nov.

EEBB86AC-FBB5-5F28-A935-634ECC2392B2

https://zoobank.org/556A1CE1-7E73-4466-9C69-F5454EB4E5CC

Fig. 14C–E

##### Type material.

***Holotype***CUMZ 13017 (Fig. 14C). Measurements: shell height 9 mm, shell width 11.6 mm and 7 whorls. ***Paratypes***CUMZ 13018 (6 shells; Fig. 14D), NHMUK (2 shells).

##### Type locality.

Limestone outcrops at Waiponla Hill, Hpa-an Township, Hpa-an District, Kayin State, Myanmar (16°56'7.4"N, 97°42'56.8"E).

##### Other material examined.

Taung Lay Cave, Hpa-an Township, Hpa-an District, Kayin State, Myanmar (17°11'40.3"N, 97°37'47.0"E): CUMZ 13019 (2 shells; Fig. 14E).

##### Diagnosis.

*Haploptychiuskarenorum* sp. nov. can be differentiated from *H.heliakosus* sp. nov. by having a convex spire, penultimate whorl angular and extended well beyond the diameter of the last whorl, and more axially deflected last whorl. In contrast, *H.heliakosus* sp. nov. possesses an elevated spire, penultimate whorl rounded, less extended beyond the diameter of the last whorl, and less axially deflected last whorl. This new species differs from *H.bombax* by having an angular penultimate whorl, more axially deflected last whorl, subcircular aperture, and broadly expanded lip. In comparison, *H.bombax* has a rounded penultimate whorl, less axially deflected last whorl, semi-ovate aperture, with thickened and slightly expanded lip. For further comparison, *H.karenorum* sp. nov. differs from *H.burmanicus*, *H.blanfordi*, and *H.thebawi* in having a more axially deflected last whorl, lower spire, penultimate whorl angular and strongly extended beyond the diameter of the last whorl, and without sinulus. The three latter species have an elevated spire, penultimate whorl rounded and slightly extended beyond the diameter of the last whorl, and less axially deflected last whorl.

##### Description.

Shell oblique-ovate, solid and translucent; whorls 6–7½; spire depressed convex and with distinct suture. Embryonic shell ~ 2½ whorls with smooth surface; following whorls growing regularly and last whorl intermediately expanded. Shell surface has moderately strong radial ridges that diminish below periphery of last whorl and around umbilicus. Penultimate whorl bluntly angular and extended beyond last whorl. Last whorl compressed to flattened, axially deflected from columellar axis. Aperture subcircular; peristome thickened, expanded, and slightly reflected. Apertural dentition with one strong parietal lamella. Umbilicus widely open and deep (Fig. 14C–E).

##### Etymology.

The specific name *karenorum* refers to the Karen people, the major ethnicity in Kayin State, Myanmar.

##### Distribution.

This species is known from two localities in the limestone karts of Kayin State, southern Myanmar.

##### Remarks.

Comparing populations from the two localities, shells of *H.karenorum* sp. nov. from the Taung Lay population (Fig. 14E) have a more elevated spire and weaker parietal lamella. However, the bluntly angular penultimate whorl and oblique ovate shells are identical to the type specimens, which we consider an intraspecific shell variation. However, these two localities are quite distant from each other, and on opposite sides of the Attaran River (Fig. 1). Thus further, genitalia information will help determine whether they are just geographical variations.

#### ﻿Genus Carinartemis

Taxon classificationAnimaliaStylommatophoraStreptaxidae

Siriboon & Panha, 2014

A3DD581A-7FE0-512D-8C6A-718F7CE6C9E2

Carinartemis Siriboon & Panha in Siriboon et al. 2014b: 166–168.

##### Type species.

*Carinartemisvesperus* Siriboon & Panha, 2014 by original designation.

##### Diagnosis.

Shell obliquely heliciform and with conical spire. Penultimate whorl keeled and extended beyond the diameter of the last whorl. Last whorl rounded to shouldered and strongly axially deflected. Aperture semi-ovate to subcircular; apertural dentition with or without one or two parietal lamellae. Genitalia with thin to thick penial sheath that covers entire penis length; penial hooks and vaginal hooks may be present.

##### Remarks.

The genus is comprised of two endemic species occurring in limestone outcrops in western Thailand. A recent phylogenetic study revealed that the previously recognized *Haploptychiuspetitii* (Gould, 1844) and *Indoartemonmedius* Siriboon & Panha, 2014 are clustered with members of the *Carinartemis* (see Siriboon et al. 2020) with strong support. Siriboon et al. (2020) suggested reassigning these two species into *Carinartemis*. Therefore, based on this phylogenetic result, the previously recognized *H.petitii* is relocated here under the genus *Carinartemis*.

#### ﻿ Carinartemis petitii

Taxon classificationAnimaliaStylommatophoraStreptaxidae

(Gould, 1844)

EA6B84AA-286D-5432-B5C3-5230C1EDE212

Fig. 17A, B

Streptaxis petitii Gould, 1844: 456, 457, pl. 24, fig. 7. Type locality: Tavoy [Dawei District, Tanintharyi Region, Myanmar].
Streptaxis petiti [sic] – Pfeiffer 1876: 8. Hanley and Theobald 1870: 4, pl. 8, fig. 4. Nevill 1878: 3. Tryon 1885: 74, pl. 14, figs 16–18. Gude 1903: 216. Kobelt 1910: 151, 152. Blanford and Godwin-Austin 1908: 4.
Haploptychius petiti [sic] – Kobelt 1906: 142, pl. 57, fig. 14. Richardson 1988: 218.
Haploptychius petitii – Siriboon et al. 2020: 7, 14, 16, fig. 3.

##### Material examined.

**Figure 17.**
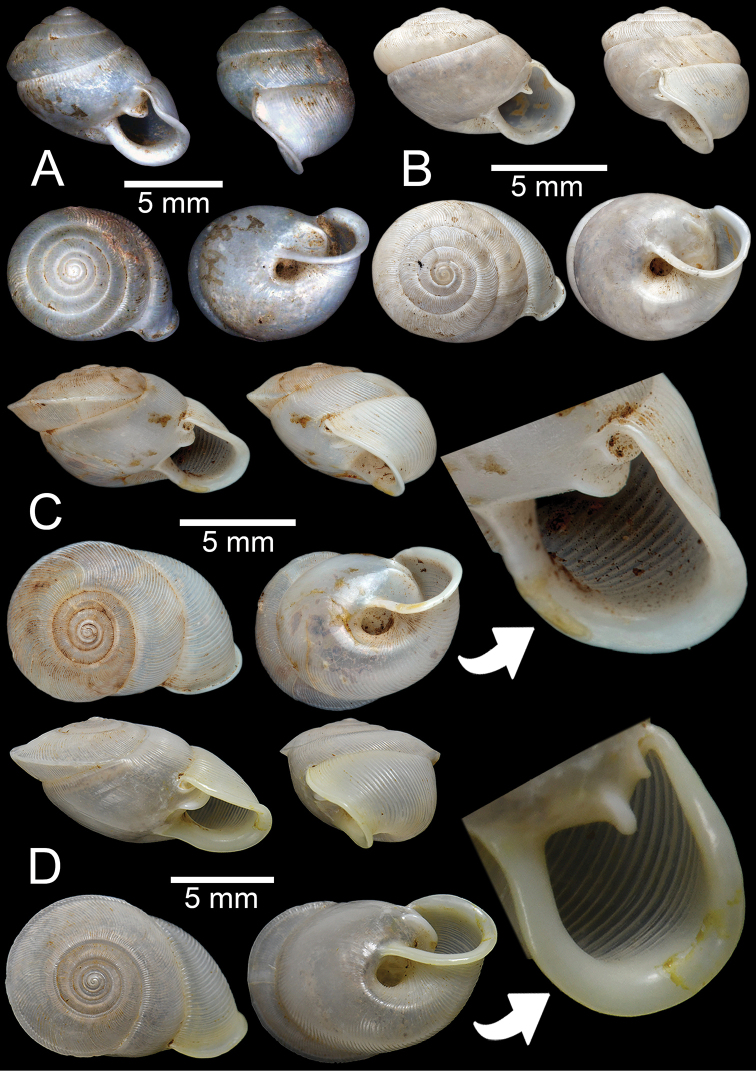
**A, B***Carinartemispetitii***A** syntype MCZ 169290 from Tavoy, British Burmah and **B** specimen NHMUK 1909.3.15.67 from Moulmein **C, D***Carinartemisexacutus***C** specimen NHMUK 1888.12.4.771–773 from Moulmein with apertural dentition and **D** specimen CUMZ 13025 from Taung Wine Cave, Hpa-an, Kayin State with apertural dentition.

***Syntype***MCZ 169290 (1 shell; Fig. 17A) from Burmah. Moulmein: NHMUK 1909.3.15.67 (2 shells, Fig. 17B) ex. Godwin-Austen collection. NHMUK 67.9.3.15 (1 shell) ex. Blanford collection.

##### Diagnosis.

*Carinartemispetitii* can be distinguished from *C.sankeyi* (Benson, 1859) by having a fine transverse ridge on the upper periphery, penultimate whorl keeled and little extended beyond the diameter of last whorl, and a subcircular aperture. In addition, *C.petitii* differs from *I.medius* from Thailand by having a more axially deflected last whorl, and only one parietal lamella present, while *I.medius* has a less axially deflected last whorl, and with one additional small palatal lamella. *Carinartemispetitii* is superficially similar to *H.blaisei* but it has an elevated spire, keeled penultimate whorl, subcircular aperture, and thicker lip.

##### Description.

Shell oblique-heliciform, white, and translucent; whorls 6½–7; spire conical with distinct suture. Shell surface glossy with fine transverse ridges, nearly smooth with a few transverse ridges near peristome. Embryonic shell ~ 2½ whorls with smooth surface; following whorls regularly coiled. Shell periphery keeled nearly the entire penultimate whorl; last whorl axially deflected. Aperture subcircular; peristome discontinuous, thickened, expanded, and reflected. Apertural dentition with one strong parietal lamella. Umbilicus open and deep (Fig. 17A, B).

##### Distribution.

This species is known from the type locality in Myanmar (Gould 1844) and Kanchanaburi Province in Thailand (Siriboon et al. 2020).

##### Remarks.

No new specimens were collected in this survey. However, the syntype (Fig. 17A) and the museum specimens (Fig. 17B) show slightly different shell forms. *Carinartemispetitii* is similar in shell form to the genus *Haplotychius* in having one parietal lamella and a deflected last whorl. However, an initial molecular analysis placed this species within the *Carinartemis* clade (Siriboon et al. 2020). This species is clearly distinct from other recognized *Carinartemis* species by its oblique-heliciform shell, translucence and a high to low conical spire with a distinct suture. Moreover, the shell surface has fine transverse ridges that diminish below periphery, whorls regularly coiled, shell periphery is keeled; umbilicus open and deep. Aperture is semi-ovate, peristome thickened, expanded, reflected, and apertural dentition with one strong parietal lamella.

#### ﻿ Carinartemis exacutus

Taxon classificationAnimaliaStylommatophoraStreptaxidae

(Gould, 1856)

202F0534-1A86-581E-B898-8C1AD67FD947

**Figure 18.**
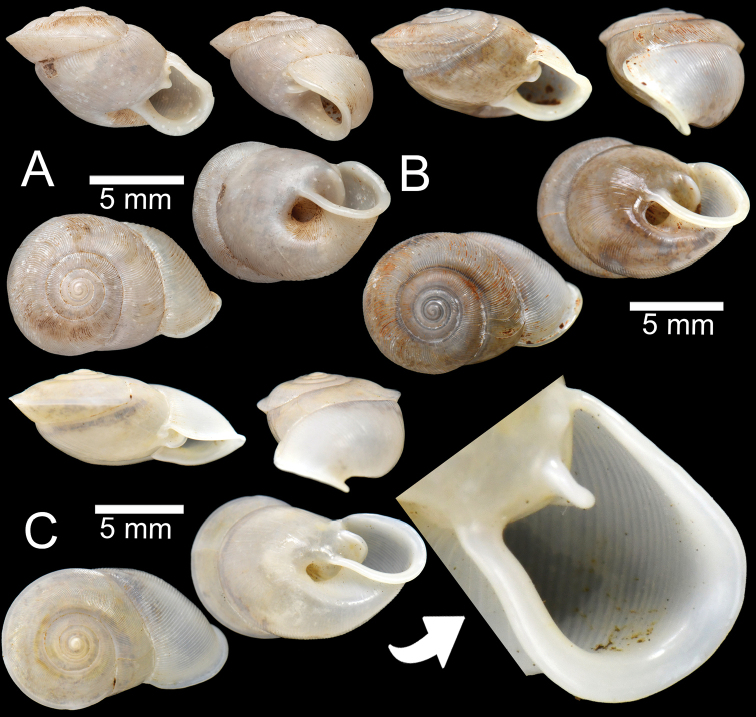
*Carinartemisexacutus***A** specimen NHMUK 1906.2.2.198 from Moulmein **B** specimen CUMZ 13021 from Bayin Nyi Cave, Hpa-an, Kayin State **C** specimen CUMZ 13023 from Lun Nga Mountain, Hpa-an, Kayin State with apertural dentition.

Figs 1 , 2D , 17C, D , 18 , 21A, B , 22 , 24D

Streptaxis exacutus Gould, 1856: 13. Type locality: Burma [Myanmar].
Streptaxis exacuta [sic] – Pfeiffer 1859: 331.
Streptaxis exacutus – Pfeiffer 1871: 30, 31, pl. 115, figs 13, 14. Hanley and Theobald 1870: 40, pl. 98, figs 8–10. Nevill 1878: 3. Tryon 1885: 72, pl. 14, figs 11, 12. Blandford and Godwin-Austin 1908: 8.
Haploptychius exacutus – Kobelt 1906: 142, 143, pl. 57, figs 16–18. Richardson 1988: 215. Siriboon et al. 2014b: 169, 171.

##### Material examined.

Moulmein: NHMUK 1874.9.3.15 (3 shells) ex. Godwin-Austen. NHMUK 1888.12.4.771–773 (3 shells; Fig. 17C). NHMUK 1906.2.2.198 (3 shells + 2 juveniles; Fig. 18A) ex. Blanford collection. NHMUK 1903.7.1.3999 (3 shells) ex. Godwin-Austen collection. Burma: NHMUK1950.12.9.170 (1 shell) ex. Laidlaw collection. Mergui: NHMUK ex. Cuming collection (2 shells). Sadhdan Cave, Hpa-an Township, Hpa-an District, Kayin State, Myanmar (16°44'23.4"N, 97°43'04.2"E): CUMZ 13020 (3 shells). Bayin Nyi Cave, Hpa-an Township, Hpa-an District, Kayin State, Myanmar (16°58'10.1"N, 97°29'30.6"E): CUMZ 13021 (8 shells; Fig. 18B), CUMZ 13022 (15 specimens in ethanol). Lun Nga Mountain, Hpa-an Township, Hpa-an District, Kayin State, Myanmar (16°44'53.2"N, 97°47'09.5"E): CUMZ 13023 (14 shells; Fig. 18C), CUMZ 13024 (50 specimens in ethanol). Taung Wine Cave, near Thiri Hpa-an Hotel, Hpa-an Township, Hpa-an District, Kayin State, Myanmar (16°50'31.1"N, 97°37'18.4"E): CUMZ 13025 (3 shells; Fig. 17D), CUMZ13026 (6 specimens in ethanol). Kaw Ka Taung Cave (Golden valley), Hpa-an Township, Hpa-an District, Kayin State, Myanmar (16°50'32.4"N, 97°37'10.9"E): CUMZ 13027 (1 shell).

##### Diagnosis.

*Carinartemisexacutus* is superficially similar to *C.sankeyi*, *C.vesperus* Siriboon & Panha, 2014, and *C.striatus* Siriboon & Panha, 2014 from western Thailand, but it has a larger shell, convex spire, immediately expanded penultimate whorl, semi-ovate aperture, two parietal lamellae, very thickened penial sheath, vas deferens passes through penial sheath, curved portion of vas deferens with very thin connective tissue, and hooks located on irregular trapezoidal penial papillae separated by longitudinal folds. In comparison, *C.sankeyi*, *C.vesperus*, and *C.striatus* have an elevated spire, regularly expanded penultimate whorl, but *C.sankeyi* has fine transverse ridges on the entire shell, subquadrangular aperture, one parietal lamella, slender atrium, thin penial sheath, vas deferens does not pass through a penial sheath, curved portion of vas deferens is without connective tissue, proximal penial hook located on laterally-flattened penial papillae, and distal penial hooks located on laterally compressed penial papillae separated by reticulated folds. In contrast, *C.vesperus* has a subcircular aperture, lacks parietal lamellae, has a less axially deflected last whorl, vas deferens passes through the penial sheath, curved portion of vas deferens is without connective tissue, and penial papillae absent. Meanwhile, *C.striatus* has strong transverse ridges over the entire shell, a semi-ovate aperture, one parietal lamella, vas deferens is attached to the distal end of the penial sheath with very thin connective tissue, and hooks are located on papillae without connected longitudinal folds.

##### Description.

Shell oblique-heliciform, white, and translucent; whorls 6–6½; spire convex with distinct suture. Shell surface glossy with fine transverse ridges, nearly smooth with few transverse ridges near peristome; varices present. Embryonic shell ~ 2½ whorls with smooth surface; following whorls intermediately coiled. Shell periphery wide and sharply keeled along nearly the entire penultimate whorl; last whorl axially deflected. Aperture semi-ovate; peristome discontinuous, thickened, expanded, and slightly reflected. Apertural dentition with one strong parietal lamella and sometimes with a second parietal lamella adjoined at a right angle. Umbilicus open and deep (Figs 17C, D, 18).

***Genital organs*.** Atrium (at) short. Penis (p) a very thin, and long tube. Penial sheath (ps) muscularly enlarged, very thickened and extending entire length of penis; penial sheath retractor muscle (psr) thin, originating near genital orifice, attached to atrium with short and thin connective tissue, and inserting distally on penial sheath (Fig. 21A). Vas deferens (vd) passes through a short portion of penial sheath, then extends to curved portion at ~ 1/11 of the penial sheath length before entering penis distally. Curved portion of vas deferens with very thin connective tissue originating at penial sheath (Fig. 21B). Penial retractor muscle (pr) thin, very long, inserting at penis and vas deferens junction.

Internal wall of atrium generally smooth with sparse atrial pores (Fig. 22A). Penial wall with scattered pale brownish penial hooks, ~ 20 hooks/200 μm^2^; hooks located on laterally flattened penial papillae and separated by longitudinal folds (Fig. 22B–D). Penial hooks small (<0.02 mm in length), expanded at base, tips pointed and curved towards genital orifice (Fig. 22E, F).

Vagina (v) very short and ~ 1/12 of penis length. Gametolytic duct (gd) a long tube extending as far as albumin gland; gametolytic sac (gs) ovate. Proximal free oviduct (fo) enlarged then tapering to smaller diameter distally. Oviduct (ov) enlarged and folded; prostate gland inconspicuous and bound to oviduct. Talon (ta) small, short and club shaped. Hermaphroditic duct (hd) bearing long seminal vesicle (sv) ~ 1/2 the length from talon to branching point of seminal vesicle (Fig. 21A).

Vaginal wall with longitudinal oblique vaginal folds, folds with nearly smooth surface and vaginal hook absent (Fig. 22G).

***Radula*.** Each row consists of 41–45 teeth with formula (22–20)–1–(20–22). Central tooth is very small with pointed cusp. Lateral and marginal teeth undifferentiated, lanceolate, unicuspid, and lanceolate. Latero-marginal teeth gradually reduced in size, with outermost teeth much smaller and shorter than inner teeth (Fig. 24D).

##### Distribution.

This species was collected from five limestone hills in Kayin State, southern Myanmar, in this survey.

##### Remarks.

This species appears at a high abundance among the limestone karsts in Hpa-an, Kayin State. All the specimens examined from the populations from Lun Nga Mountain and Bayin Nyi Cave have a very small to indistinct upper parietal lamella, and some specimens from Bayin Nyi Cave have strong transverse ridges on almost the entire whorl. Moreover, the Lun Nga Mountain population have a last whorl that is more extended anteriorly and an elongated semi-ovate aperture (Fig. 18C). However, the genitalia in all localities show no difference from this locality; thus, we consider them as intraspecific variations within *C.exacutus*. Some specimens from Bayin Nyi Cave have strong transverse ridges on almost the entire whorl.

Although the type locality was listed as ‘Burma’ [Myanmar], all the known records and specimens examined in this study were collected from Kayin State. Therefore, the precise type locality of this species is probably southwestern Myanmar in Mon State and Kayin State.

#### ﻿ Carinartemis sankeyi

Taxon classificationAnimaliaStylommatophoraStreptaxidae

(Benson, 1859)

A9B3BC26-1C09-572F-A717-DFAC7ACCC058

**Figure 19.**
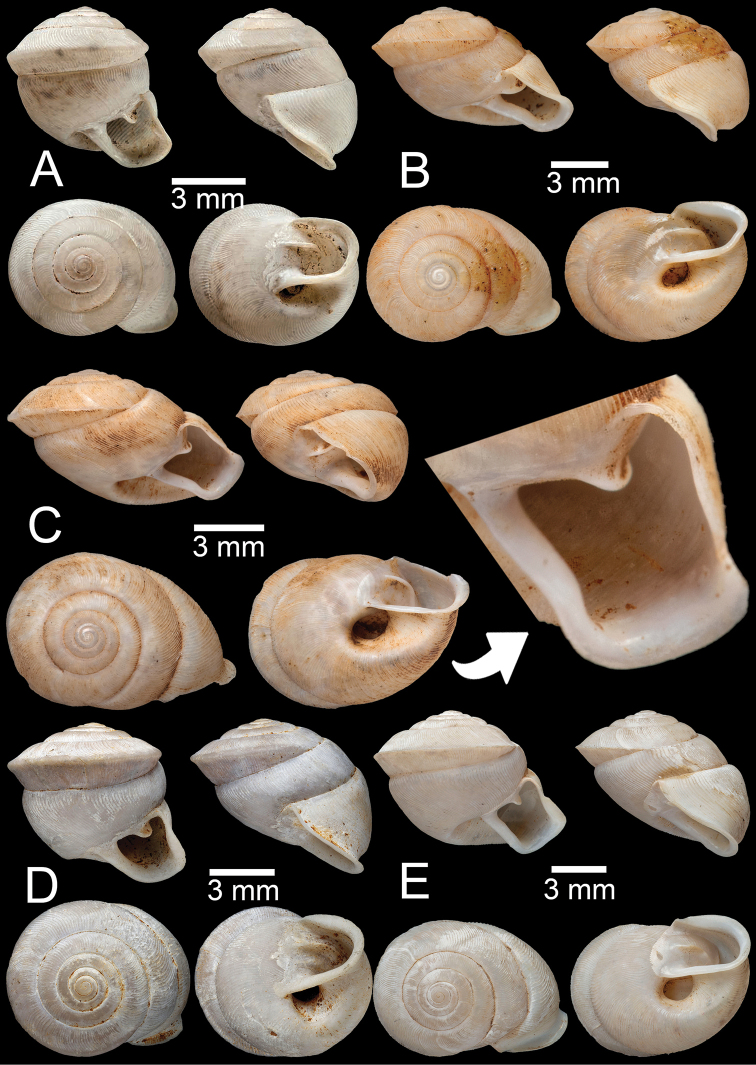
*Carinartemissankeyi***A** syntype UMZC I.102740 from Moulmein **B** specimen NHMUK 1888.12.4.792–794 from Moulmein **C** specimen NHMUK 1906.2.2.341 from Moulmein with apertural dentition **D** specimen NHMUK 1871.9.23.61 from Moulmein **E** specimen NHMUK ex. Cuming collection from Mergui.

**Figure 20.**
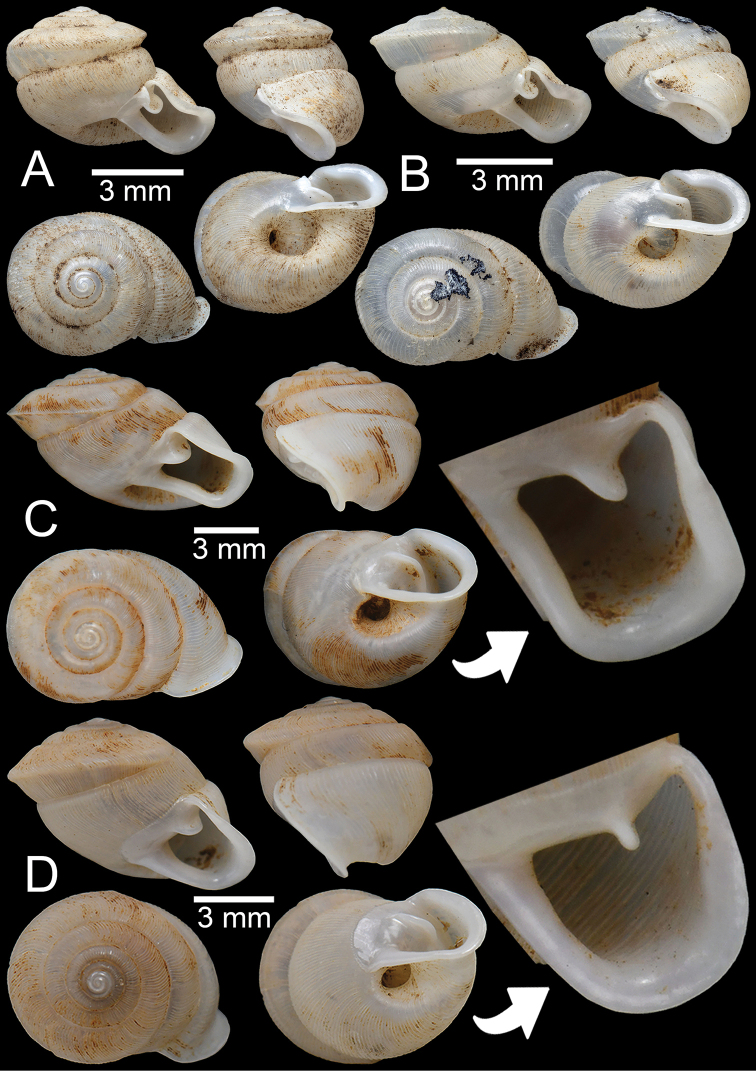
*Carinartemissankeyi***A** specimen NHMUK 1903.7.1.4001 from Moulmein **B** specimen NHMUK 1888.12.4.781–783 from Kwengan Hill **C** specimen CUMZ 13031 from Dhammatat Cave, Mawlamyine, Mon State with apertural dentition **D** specimen CUMZ 13029 from Kayon Cave, Mawlamyine, Mon State with apertural dentition.

**Figure 21.**
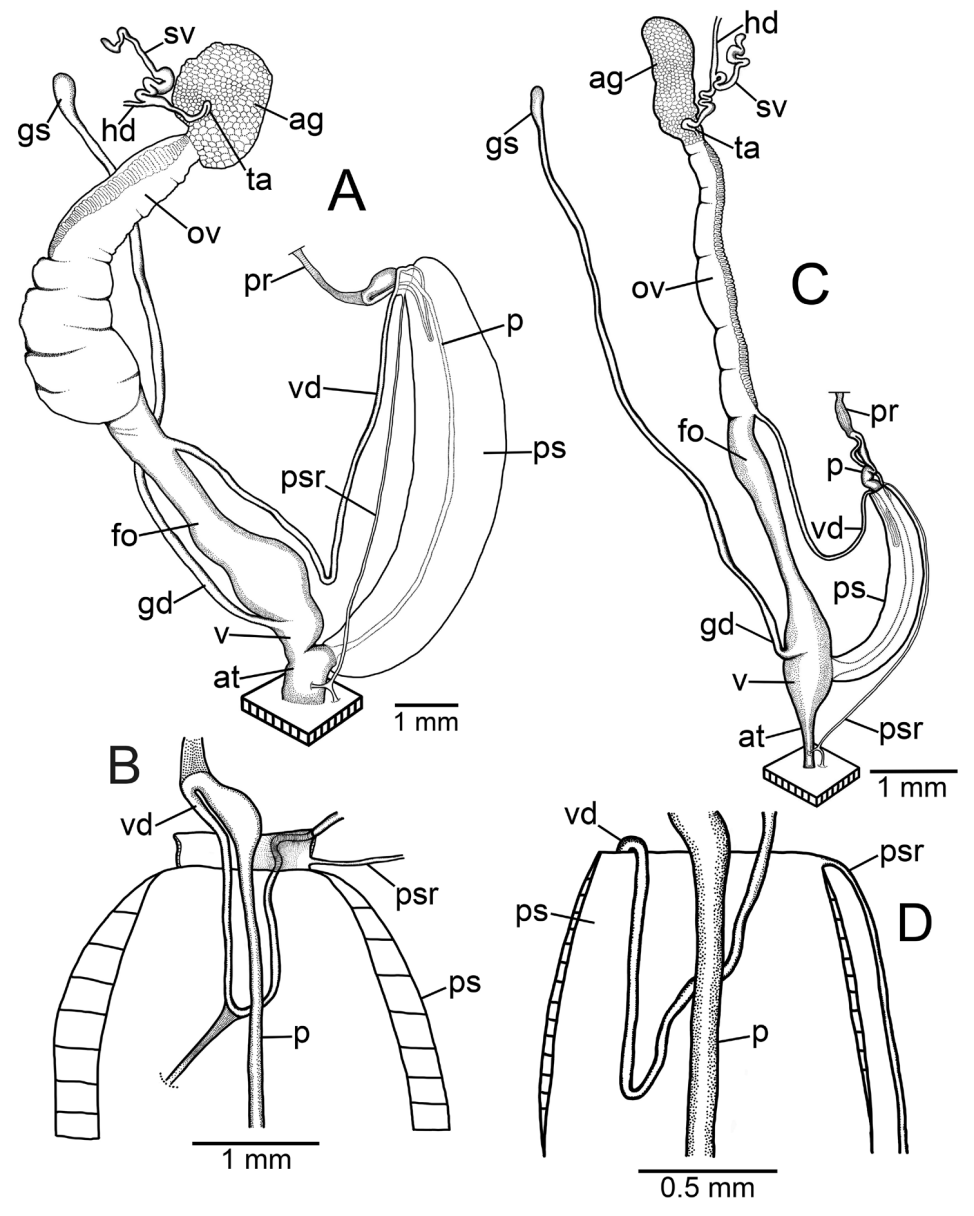
Genital anatomy of **A, B***Carinartemisexacutus*, specimen CUMZ 13021 **A** reproductive system and **B** insertion of vas deferens into penial sheath **C, D***Carinartemissankeyi*, specimen CUMZ 13029 **C** reproductive system and **D** insertion of vas deferens into penial sheath.

Figs 1 , 2E, F , 19 , 20 , 21C, D , 23 , 24E, F

Streptaxis sankeyi Benson, 1859a: 472. Type locality: Moulmein [Mawlamyine Township, Mon State, Myanmar]. Pfeiffer 1868: 442. Hanley and Theobald 1870: 4, pl. 8, fig. 7. Tryon 1885: 72, pl. 14, figs 2, 3. Blanford and Godwin-Austen 1908: 8, fig. 7.
Streptaxis sankeyanus Stoliczka, 1871: 167, 168, pl. 7, fig. 14 (unjustified emendation). Hanley and Theobald 1870: 4, pl. 8, fig. 7. Nevill 1878: 3. Tryon 1885: 72, pl. 14, figs 9, 10.
Streptaxis hanleyanus Stoliczka, 1871: 168, 169, pl. 7, fig. 15. Type locality: Prope Moulmein, ad flumen Attaran [Attaran River, Mawlamyine, Mon State, Myanmar]. Blanford and Godwin-Austen 1908: 8, 9.
Haploptychius sankeyi – Kobelt 1906: 147, pl. 57, figs 11, 12, pl. 62, figs 6, 7. Richardson 1988: 220. Siriboon et al. 2014b: 169.
Oophana hanleyana – Richardson 1988: 235.

##### Material examined.

***Syntype*** UMZC I.102740 (6 shells; Fig. 19A) from Moulmein. Moulmein: NHMUK 1954.6.3.556 (1 shell) ex. Hawkins collection. NHMUK 1906.2.2.341 (4 shells; Fig. 19C) ex. Blanford collection. NHMUK 1872.9.3.15 (5 shells) ex. Godwin-Austen collection. NHMUK 1903.7.1.4001 (1 shell; Fig. 20A) ex. Godwin-Austen collection. NHMUK Acc. No. 1733 ex. Oldham collection (2 shells). NHMUK 1888.12.4.792–794 (3 shells; Fig. 19B). NHMUK 1871.9.23.61 (1 shell; Fig. 19D). Paboung Toung, Burma: NHMUK 1891.3.17.577–578 (2 shells) ex. Hungerford collection. Mergui: NHMUK 20210051 (1 shell; Fig. 19E) ex. Cuming collection. Kwengan Hill: NHMUK 1888.12.4.781–783 (3 shells; Fig. 20B) ex. Blanford collection. Saddan Cave situated on same karsts ~ 600 m south of Kayon Cave, Mawlamyine Township, Mawlamyine District, Mon State, Myanmar (16°31'42.8"N, 97°43'2.1"E): CUMZ 13028 (7 shells). Kayon Cave [previously known as Farm Caves] ~ 10 km from Mawlamyine Township, Mawlamyine District, Mon State, Myanmar (16°32'0.5"N, 97°42'53.5"E: CUMZ 13029 (13 shells; Fig. 20D), CUMZ 13030 (5 specimens in ethanol). Dhammatat Cave, Mawlamyine Township, Mon State, Myanmar (16°30'23.0"N, 97°48'36.3"E): CUMZ 13031 (12 shells; Fig. 20C).

##### Diagnosis.

*Carinartemissankeyi* is superficially similar to *C.vesperus* and *C.striatus* in having a subquadrangular aperture, and less expanded and continuous peristome, slender atrium, vas deferens does not pass through a penial sheath, proximal penial hooks located on laterally flattened penial papillae, and distal penial hooks located on laterally compressed penial papillae separated by reticulated folds. In contrast, the shell of *C.vesperus* has fine transverse ridges (nearly smooth) with few transverse ridges near peristome, varices absent, periphery more extended beyond the diameter of last whorl, lacking parietal lamella, and penial papillae absent. Meanwhile, *C.striatus* has vas deferens attached to the distal end of penial sheath with very thin connective tissue, and hooks located on papillae without connected longitudinal folds.

##### Description.

Shell oblique-heliciform, white, translucent; whorls 6–7; spire conical with distinct suture. Shell surface glossy with fine transverse ridge across the entire shell; varices present. Embryonic shell ~ 2½ whorls with smooth surface; following whorls regularly coiled. Shell periphery wide and sharply keeled around nearly the entire penultimate whorl; last whorl axially deflected. Aperture subquadrangular; peristome continuous, thickened, expanded, and reflected. Apertural dentition with one strong parietal lamella. Umbilicus open and deep (Figs 19, 20).

***Genital organs*.** Atrium (at) short, thin and slender. Penis (p) very thin and long. Penial sheath (ps) thin and extending nearly entire length of penis; penial sheath retractor muscle (psr) thin, originating near genital orifice and attached to atrium with short and thin connective tissue, and inserting distally on penial sheath (Fig. 21C). Vas deferens (vd) runs downwards to curved portion, ~ 1/3 of the penial sheath length, without insertion before entering penis distally (Fig. 21D). Penial retractor muscle (pr) thin, very long, inserting at penis and vas deferens junction.

**Figure 22.**
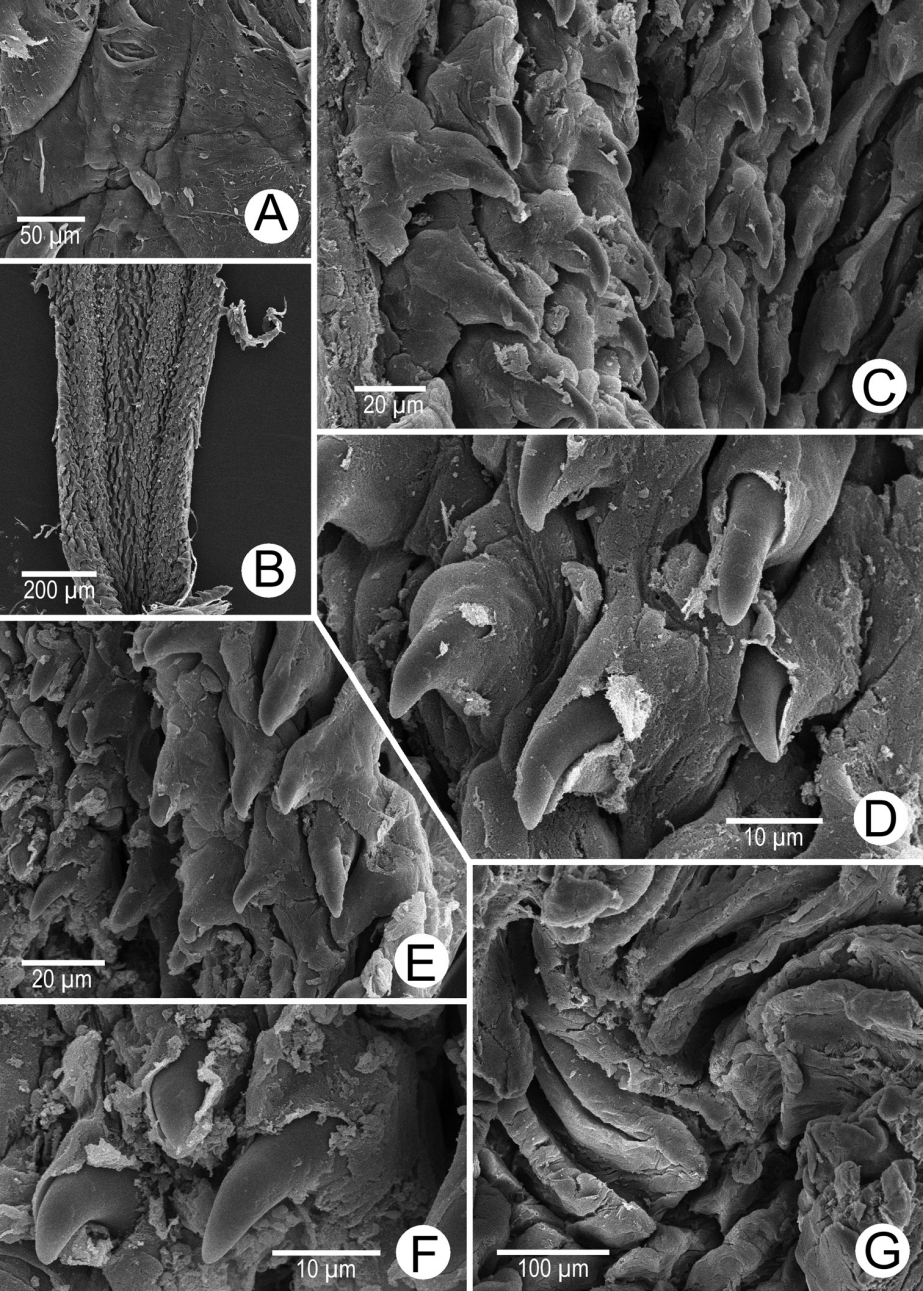
Internal sculpture of genitalia of *Carinartemisexacutus*, specimen CUMZ 13021 **A** atrium surface **B** overview of internal penial wall **C** arrangement of penial hooks on proximal part of penis **D** lateral view of penial hooks **E** arrangement of penial hooks on middle part of penis **F** lateral view penial hooks **G** arrangement of longitudinal oblique vaginal folds.

**Figure 23.**
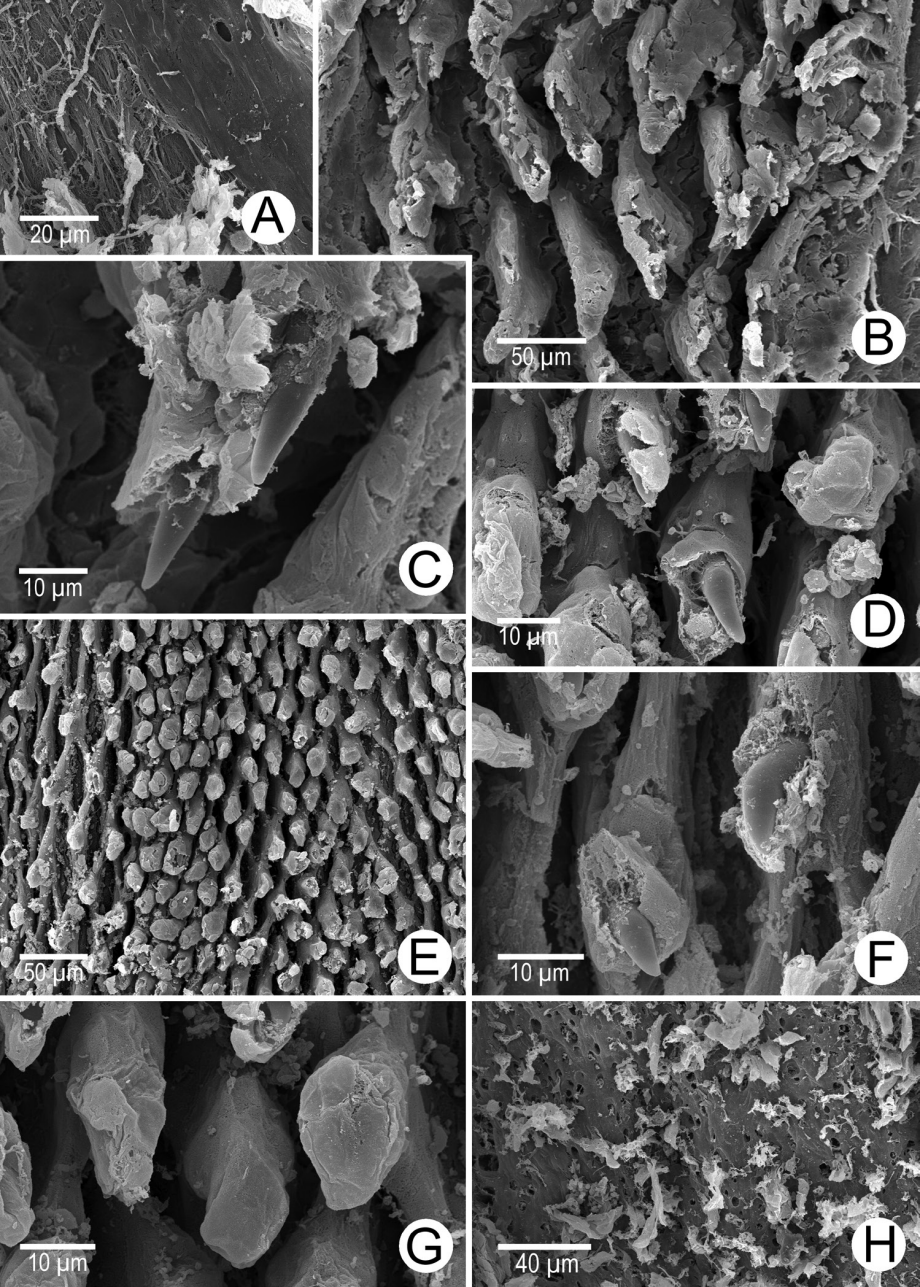
Internal sculpture of genitalia of *Carinartemissankeyi*, specimen CUMZ 13029 **A** atrium surface **B** arrangement of penial hooks on proximal part of penis **C** lateral view of exposed proximal penial hooks **D** penial hooks on middle part of penis **E** arrangement of penial hooks on distal part of penis **F** uncovered distal penial hooks with pointed tip **G** distal penial hooks embedded in penial papillae **H** vaginal surface with vaginal pores.

Internal wall of atrium generally smooth with sparse atrial pores (Fig. 23A). Proximal penial wall covered with scattered and pale brownish penial hooks, ~ 12 hooks/200 μm^2^. Proximal penial hooks located on laterally flattened penial papillae; hooks small and short (< 0.03 mm in length), slightly expanded at base, tips obtuse and curved towards genital orifice (Fig. 23B, C). Middle and distal penial walls densely covered with pale brownish hooks, ~ 20 hooks/200 μm^2^. Middle and distal hooks located on laterally compressed penial papillae separated by reticulated folds; hooks small, short (<0.01 mm in length), slightly expanded at base, and tips pointed (Fig. 23D–G).

Vagina (v) short, stout and ~ 1/5 of penis length. Gametolytic duct (gd) a long tube extending as far as albumin gland; gametolytic sac (gs) ovate. Proximal free oviduct (fo) enlarged then tapering to smaller diameter in middle section, and slightly enlarged distally. Oviduct (ov) enlarged and folded; prostate gland inconspicuous and bound to oviduct. Talon (ta) small, short and club shaped. Hermaphroditic duct (hd) bearing long seminal vesicle (sv) ca. the same length as from talon to branching point of seminal vesicle (Fig. 21C).

Vaginal wall generally smooth with vaginal pores, and vaginal hook absent (Fig. 23H).

##### Distribution.

This species occurs from three localities in Mon State, southern Myanmar and is likely to be endemic to this area.

##### Radula.

**Figure 24.**
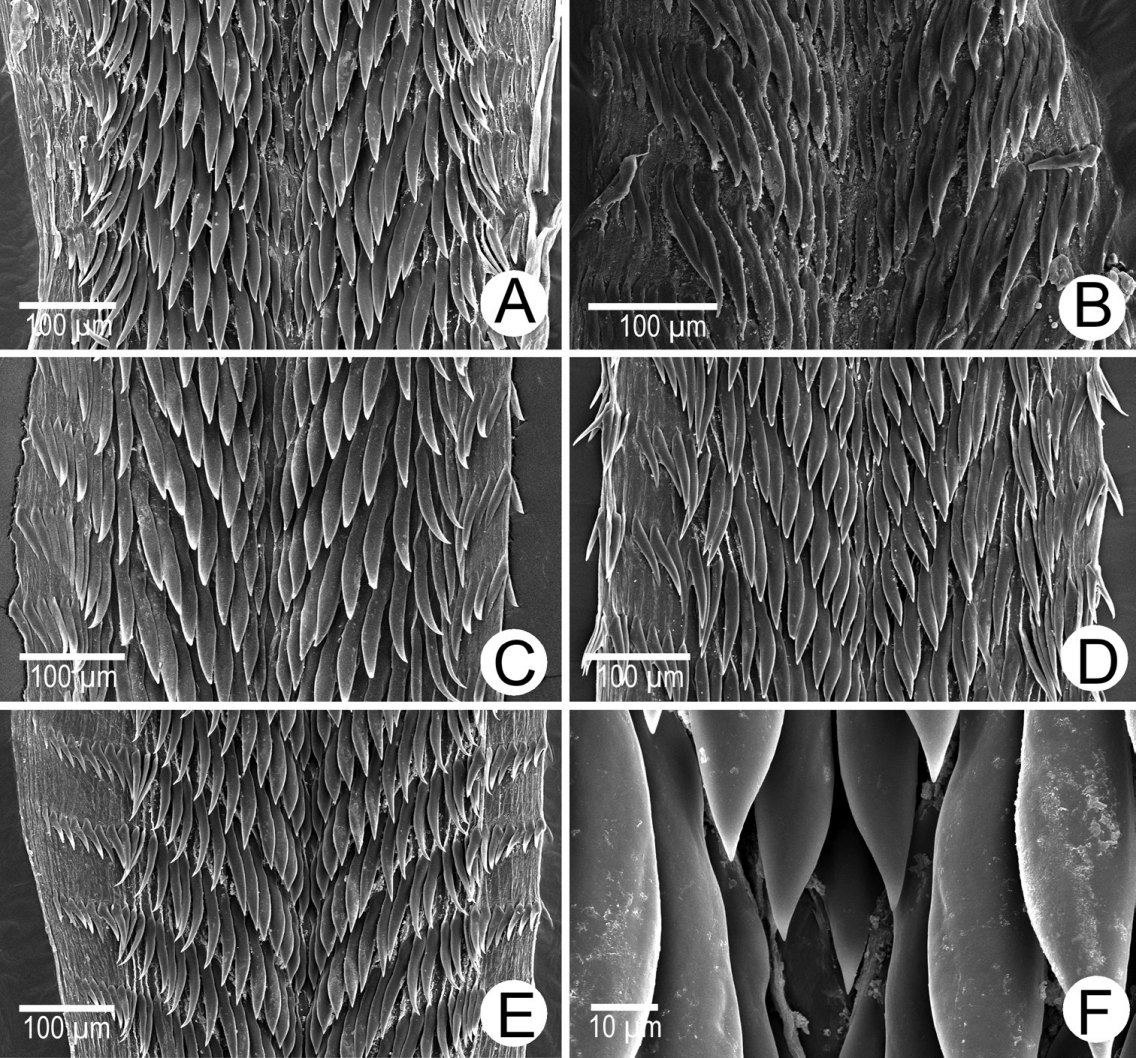
Radula of specimens **A***Discartemontonywhitteni*, paratype CUMZ 13001 **B***Discartemonpaurodeviatus* sp. nov., paratype CUMZ 13003 **C***Haploptychiusheliakosus* sp. nov., paratype CUMZ 13014 **D***Carinartemisexacutus*, specimen CUMZ 13021 **E, F***Carinartemissankeyi*, specimen CUMZ 13029 **E** overview of radula and **F** high magnification of central teeth.

Each row consists of 43–49 teeth with formula (21–24)–1–(21–24). Central tooth small with pointed cusp. Lateral and marginal teeth undifferentiated, unicuspid and lanceolate. Latero-marginal teeth gradually reduced in size, with outermost teeth much smaller and shorter than inner teeth (Fig. 24D).

##### Remarks.

Benson (1859a) introduced *C.sankeyi* based on a single specimen from ‘Moulmein’. Later, Stoliczka (1871) provided some diagnostic characters with a description of the soft body color and suggested that the Farm-Cave is possibly the correct type locality. This historical locality name refers to a group of caves located on a karst ridge, namely Kayon Hill, situated on the west bank of the Attaran River. At the same time, Stoliczka (1871) proposed *Streptaxishanleyanus* based on a single specimen with a small and depressed shell, wide umbilicus, and almost rectangular aperture. However, this holotype specimen was collected from the same geographical area as *C.sankeyi*. Therefore, we consider this nominal species as a smaller shell form and so treat it as a junior synonym with *C.sankeyi*.

The original spelling of this nominal species was *sankeyi*, which was intentionally modified to *sankeyanus* by Stoliczka (1871) without a clear reason. Therefore, this unjustified emendation name was made available with its authorship and date and became a junior objective synonym (ICZN 1999: Arts 32.3, 33.2.3, 50.5).

Recently, the population collected from Dhammatat Cave showed a more rectangular aperture, slightly compact penultimate whorl, and larger parietal lamella (Fig. 20C), but this species could only be examined from the empty shells. Therefore, we have to conclude that these differences are an intraspecific variation until more specimens are available, or at least the genitalia.

## ﻿Discussion

This research presents all known Streptaxidae in Myanmar, comprising eighteen species belonging to five genera (*Carinartemis*, *Discartemon*, *Haploptychius*, *Oophana*, and *Perrottetia*), including fourteen formerly known species and four new species (Table 2). In addition, this study has added the first anatomical information for three formerly known species (*D.tonywhitteni*, *C.exacutus*, and *C.sankeyi*) and one new species (*H.heliakosus* sp. nov.). The diversity yielded from the southern part of Myanmar (four genera with nine species) was higher than in the central-northeast part (two genera with two species). However, the number of streptaxid taxa found in the central-northeast part of Myanmar are believed to be low because sampling could only be conducted in a few areas. Further land snail surveys should also focus on areas we could not cover in this expedition, such as Rakhine State and Bago Region, areas that contain many of the formerly known species (Theobald 1864; Blanford 1865).

**Table 2.** Comparative shell morphology of all recognized streptaxid species in Myanmar.

Species	Shell shape	Spire	Shell surface	Penultimate whorl	Last whorl	Apertural dentition (lamellae)
***D.tonywhitteni***	depressed heliciform	low-conical to convex	glossy with fine transverse ridges	flattened	angular (sometimes narrowly rounded)	one parietal, one palatal, one basal, one columellar, and one supracolumellar (sometimes with small upper palatal)
***D.paurodeviatus* sp. nov.**	globose heliciform	conical	glossy with fine transverse ridges	rounded, not extended beyond last whorl	rounded, little axially deflected	one parietal, one palatal, one basal, and one columellar (sometime with supracolumellar)
***O.elisa***	oblique heliciform	convex	transverse ridges	angular, extended beyond last whorl	axially deflected	one parietal, one palatal, one basal, and one columellar (sometimes with supracolumellar)
***O.mouhoti***	oblique ovate	elevated conical	glossy with fine transverse ridges	slightly angular, not extended beyond last whorl	axially deflected	one parietal, one upper palatal, one palatal, and one basal
***O.obtusus***	oblique ovate	convex	glossy with fine transverse ridges	rounded to weakly angular and scarcely extended beyond last whorl	slightly axially deflected	one parietal, one palatal, and one columellar
***O.laevis***	oblique heliciform	low convex	glossy with transverse ridges	rounded, not extended beyond last whorl	axially deflected	one parietal, and one basal, (columellar lamella present or absent)
***P.theobaldi***	sub-oblique heliciform	convex	glossy with transverse ridges	rounded, not extended beyond last whorl	axially deflected	one parietal nearly adjoint with small second parietal, one upper palatal, one palatal, one basal, and one bifid columellar
***H.bombax***	oblique ovate	convex	glossy with fine transverse ridges	rounded, slightly extended beyond last whorl	axially deflected	only one parietal lamella
***H.blanfordi***	oblique heliciform	convex	glossy with fine transverse ridges	rounded, slightly extended beyond last whorl	axially deflected	one parietal (sometimes with small palatal)
***H.burmanicus***	oblique heliciform	convex	glossy with fine transverse ridges	rounded, slightly extended beyond last whorl	axially deflected	one parietal
***H.solidulus***	oblique ovate	elevated conical	glossy with fine transverse ridges	slightly angular, not extended beyond last whorl	axially deflected	one parietal
***H.thebawi***	oblique heliciform	conical	glossy with transverse ridges	rounded, less extended beyond last whorl	axially deflected	one parietal
***H.tenasserimicus* sp. nov.**	sub-oblique heliciform	low convex	glossy with fine transverse ridges	angular, extended beyond last whorl	axially deflected	one parietal
***H.heliakosus* sp. nov.**	oblique ovate	low conical	glossy with transverse ridges	rounded, extended beyond last whorl	axially deflected	one parietal
***H.karenorum* sp. nov.**	depressed ovate	depressed-convex	moderately strong transverse ridges	bluntly angular, extended beyond last whorl	axially deflected	one parietal
***C.petitii***	oblique heliciform	high to low conical	fine transverse ridges	subangular, slightly extended beyond last whorl	axially deflected	one parietal
***C.exacutus***	oblique heliciform	convex	glossy with fine transverse ridges	sharply keel, extended beyond last whorl	axially deflected	one parietal (sometimes with second parietal)
***C.sankeyi***	oblique heliciform	convex	glossy with fine transverse ridges	sharply keeled, extends beyond last whorl	axially deflected	only one parietal

Most reported streptaxids in Myanmar occupy a narrow distribution, and some species were found in only a single area, such as one species of *Perrottetia* in Shan State, two species of *Discartemon* in the Tanintharyi Region, and two species of *Carinartemis* in Kayin and Mon States, which are adjacent regions. In contrast, *Haploptychius* has a wide distribution, ranging from central-northeastern to southern Myanmar, including previously recorded localities, such as Rakhine State and the Bago Region (Theobald 1864; Blanford 1865). Notably, the shell size of streptaxids from the northern part of Myanmar is smaller than those from the southern part, and the population density is also lower. In southern regions, geomorphological and climatic conditions seem to offer more favorable habitats for snails (Siriboon et al. 2020; Sutcharit et al. 2020). On the other hand, this may reflect very narrow distribution ranges, naturally low population levels, or difficulty in finding the northern species due to their smaller size. The two species from the genus *Discartemon* are clearly different in shell morphology, genital structure, and geographic distribution. *Discartemontonywhitteni* is distributed on the mainland, while *D.paurodeviatus* sp. nov. occurs on an isolated island. *Discartemon* species are mainly localized in southern Thailand and Peninsular Malaysia (Siriboon et al. 2014a, 2020).

Herein, all previously documented species of *Oophana* have been redescribed, based on original descriptions compared with historical museum specimens except for *O.mouhoti*, a new record in Myanmar but based on only a single shell. Siriboon et al. (2020) reported this species from several localities in western Thailand, adjacent to Mon State and Kayin State, but it seems rare in Myanmar even though most the historical materials are recorded from Mawlamyine and Tanintharyi. A phylogenetic analysis revealed that *O.mouhoti* and *O.strangulatus* (von Möllendorff, 1894) were members of the same clade, and were distinct from other congeners in having a more cylindrical shell. In comparison to the Thailand species, most *Oophana* species from Myanmar show a more elongated globose-ovate shell form and weaker apertural dentition (see Siriboon et al. 2020), but *O.elisa* is still ambiguous. Genetically, *Oophana* from Myanmar appear to be closer to the *O.mouhoti* clade and have a similar shell shape and apertural dentition; however, the genital anatomy of the *Oophana* species remains largely unknown.

The most dominant genus, *Haploptychius*, are found mainly in Hpa-an, in the southern part of Myanmar. This genus also shows variability in its shell shape related to their geographic distribution. For example, snails from the (i) Kayin State have more globosely ovate shells and a larger size, while those from (ii) Tanintharyi Region have depressed shells, and those from (iii) Mandalay Region and Shan State have smaller, ovate shells and most bear only one parietal lamella. Exceptionally, *H.heliakosus* sp. nov. from the type population has an extra small tubercle next to the parietal lamella, and similarly, *H.blanfordi* has a small palatal lamella. While the male reproductive organ of the *Haploptychius* species in Laos shows a relatively thin penial sheath with slender, elongate penial hooks (Inkhavilay et al. 2016), surprisingly, *H.heliakosus* sp. nov. and *C.exacutus* superficially share a lack of differentiation in their external genitalia features by having a very thickened penial sheath that covers the entire penis, with broader basal penial hooks, and obvious penial papillae (Table 2). Further molecular analysis may suggest the possible cause of these shared characteristics among the streptaxids.

So far, five species in the genus *Carinartemis* have been recorded from Thailand and Myanmar (Siriboon et al. 2014b), of which four species have been investigated in terms of their genitalia. The distinctive traits of the male organs of *Carinartemis* from Myanmar are the possession of penial hooks with reticulated folds that are covered with penial papillae (Table 2). The internal sculpture of the genitalia of *C.exacutus* and *C.sankeyi* could be useful in discriminating the species, although their shell morphology is highly similar, but distinct from the Thai species (Siriboon et al. 2014b). In addition, the shell shape shared among *Carinartemis* species was observed in *H.karenorum* sp. nov. and *H.tenasserimicus* sp. nov., which tend to have an angular penultimate whorl that extends beyond the last whorl. This angular or slightly keeled penultimate whorl also occurs in some species of *Oophana* and *Indoartemon* (Siriboon et al. 2020). As Siriboon et al. (2020) suggested, to stabilize the traditional taxonomic boundary in these groups, more information on at least the genitalia is required. Furthermore, these records of new biological diversity in Myanmar indicate the uniqueness of its ecosystems and should promote more discussion concerning conservation efforts in Myanmar.

## Supplementary Material

XML Treatment for Discartemon

XML Treatment for Discartemon tonywhitteni

XML Treatment for Discartemon paurodeviatus

XML Treatment for Oophana

XML Treatment for Oophana elisa

XML Treatment for Oophana mouhoti

XML Treatment for Oophana obtusus

XML Treatment for Oophana laevis

XML Treatment for Perrottetia

XML Treatment for Perrottetia theobaldi

XML Treatment for Haploptychius

XML Treatment for Haploptychius bombax

XML Treatment for Haploptychius blanfordi

XML Treatment for Haploptychius burmanicus

XML Treatment for Haploptychius solidulus

XML Treatment for Haploptychius thebawi

XML Treatment for Haploptychius tenasserimicus

XML Treatment for Haploptychius heliakosus

XML Treatment for Haploptychius karenorum

XML Treatment for Carinartemis

XML Treatment for Carinartemis petitii

XML Treatment for Carinartemis exacutus

XML Treatment for Carinartemis sankeyi
